# Integration of proteome and transcriptome refines key molecular processes underlying oil production in *Nannochloropsis oceanica*

**DOI:** 10.1186/s13068-020-01748-2

**Published:** 2020-06-18

**Authors:** Wuxin You, Li Wei, Yanhai Gong, Mohamed El Hajjami, Jian Xu, Ansgar Poetsch

**Affiliations:** 1 Single-Cell Center CAS Key Laboratory of Biofuels and Shandong Key Laboratory of Energy Genetics, Qingdao Institute of Bioenergy and Bioprocess Technology, Chinese Academy of Sciences, Qingdao, Shandong China; 2 Department of Plant Biochemistry, Ruhr University Bochum, Bochum, Germany; 3 University of Chinese Academy of Science, Beijing, China; 4 Laboratory for Marine Biology and Biotechnology, Qingdao National Laboratory for Marine Science and Technology, Qingdao, 266237 China; 5 College of Marine Life Sciences, Ocean University of China, Qingdao, 266003 China

**Keywords:** Triacylglycerols, Oleaginous algae, Photosynthesis, Lipid biosynthesis

## Abstract

**Background:**

Under nitrogen deficiency situation, *Nannochloropsis* spp. accumulate large amounts of lipids in the form of triacylglycerides (TAG). Mechanisms of this process from the perspective of transcriptome and metabolome have been obtained previously, yet proteome analysis is still sparse which hinders the analysis of dynamic adaption to nitrogen deficiency. Here, proteomes for 3 h, 6 h, 12 h, 24 h, 48 h and 10th day of nitrogen deplete (N−) and replete (N+) conditions were obtained and integrated with previous transcriptome data for *N. oceanica*.

**Results:**

Physiological adaptations to N− not apparent from transcriptome data were unveiled: (a) abundance of proteins related to photosynthesis only slightly decreased in the first 48 h, indicating that photosynthesis is still working efficiently, and protein amounts adjust gradually with reduction in chloroplast size. (b) Most proteins related to the TCA cycle were strongly upregulated after 48 h under N−, suggesting that respiration is enhanced after 48 h and that TCA cycle efflux supports the carbon required for lipid synthesis. (c) Proteins related to lipid accumulation via the Kennedy pathway increased their abundance at 48 h, synchronous with the previously reported diversification of fatty acids after 48 h.

**Conclusions:**

This study adds a proteome perspective on the major pathways for TAG accumulation in *Nannochloropsis* spp. Temporal changes of proteome exhibited distinct adaptation phases that are usually delayed relative to transcriptomic responses. Notably, proteome data revealed that photosynthesis and carbon fixation are still ongoing even after 48 h of N−. Moreover, sometimes completely opposite trends in proteome and transcriptome demonstrate the relevance of underexplored post-transcriptional regulation for N− adaptation.

## Background

Under nitrogen deplete (N−) condition, *Nannochloropsis* spp. accumulate lipid in the form of triacylglycerides (TAG) that can make up to 60% of its dry weight [1, 2]. As promising organisms for the renewable production of biodiesel and various other products, *Nannochloropsis* spp. have played an important role in biotechnology [3–5]. A general drawback for industrial application of *Nannochloropsis spp.* is the slowed growth under N−. Thus, an understanding of TAG biosynthesis pathways and their induction under N− is paramount to optimize its biofuel production. TAG synthesis in *Nannochloropsis* is thought to be similar to vascular plants and occurs via two pathways [6]: (a) Kennedy pathway dependent on acyl-CoA and (b) the phospholipid diacylglycerol acyltransferase (PDAT) mediated alternative pathway, which uses fatty acids from lipids turnover. Intriguingly, adaptation to N− displayed discrete temporal regulation for enzymes in Kennedy pathway and the alternative pathway [6]. Involved enzymes are associated with different subcellular compartments and harbor diverse physiological functions [7]. The diacylglycerol acyltransferases (DGAT) in the Kennedy pathway, responsible for the formation of TAG, are suspected pacemakers of TAG synthesis [4, 8]. In *N. oceanica* IMET1, there are eleven isoforms of DGATs that originated, respectively, from green alga, red alga, and eukaryotic hosts [9]. An elaborate regulation of TAG synthesis is suggested by their gene dose being several times larger than in higher plants and animals [9, 10]. In fact, multiple species of *Nannochloropsis* have been sequenced [9–11] and transcriptomic analyses conducted [10, 12, 13]. A time-series transcriptomic and lipidomic dataset tracking IMET1 in N− revealed increased transcript abundance for seven putative DGAT isoforms [6]. In addition, other genes important for lipid synthesis as well as genes for the provision of carbon precursors and energy for the de novo fatty acid synthesis were upregulated. On the lipidomic level, lipids constituting chloroplast membrane were reduced after the N− [6]. Concurrently, the TAGs stored in the lipid body accumulated over time [14], indicating that part of the chloroplast membrane was converted to TAG. Moreover, the structural diversity of TAG increased dramatically after 24 h of N− [6]. Moreover, analysis of transcription factors (TF) resulted in 34 TFs (with 11 TFs potentially involved in the TAG biosynthesis pathway), 30 TF binding site motifs and 2368 regulatory connections between TFs and targets [15]. Furthermore, metabolic changes for protein, starch and TAG content monitored on the single-cell level in IMET1 were characterized with single-cell Raman spectra, which provide a powerful tool to screen strains or profile bioprocess [16].

In the previous -omics researches, emphasis was on lipid-related pathways [6, 17, 18]. So far, few proteome studies are available for *Nannochloropsis* species under N−. Initial proteome analyses of the strain *Nannochloropsis oceanica* IMET1 employing 2D-PAGE resulted in the detection of 1487 proteins spots [19] that indicated nitrogen recycling and storage of lipids. Another proteome analysis identified around 50 proteins that significantly changed via MALDI-TOF [20]. N− led to substantial decrease in total cellular protein amount and lower abundance of proteins involved in photosynthesis, carbon fixation and chlorophyll biosynthesis [19]. Limitations of these studies are incomplete coverage of proteome and sampling over the adaptation time. Furthermore, no comparison of proteome with transcriptome and metabolome under similar cultivation conditions has been attempted for *N. oceanica*, this contrasts with *C. reinhardtii,* where N-deprivation has already been studied by integrated -omics analyses [21, 22].

*Nannochloropsis oceanica* IMET1 is an industrial oleaginous strain that can accumulate TAG up to 69% (dry matter) lipids [23] under N-deprivation. This is a striking difference physiological from *C. reinhardtii,,* which mostly accumulates starch in this situation [16, 24]. Tools for strain engineering and single-cell based rapid phenotyping have been developed for this strain (e.g., CRISPR/Cas9 and ramanome analysis [23–28]). Moreover, cultivation methods have been optimized in large-scale bioreactors [3, 29] and lipid extraction [30]. These efforts established this strain as one model of strain engineering for microalgae based carbon fixation [25, 31, 32] and TAG synthesis [33, 34]. Therefore, here employing *N. oceanica* IMET1 as a model, we tracked the proteome profiles from 3 h, 6 h, 12 h, 24 h, 48 h up to 10 days during microalgal adaption to N−. These data were integrated with transcriptome [6] and other data from former research to unveil regulatory hierarchies. It was concluded that several important adaptations (e.g., photosynthetic machinery) are only sufficiently explained by proteomics, but not transcriptomics. Altogether, this work provided novel insight into the adaptation process and its regulation. Such improved understanding of important nodes of the *N. oceanica* metabolic and regulatory network may serve in strain engineering to further enhance carbon fixation and TAG production.

## Results

### On the proteome level, the temporal adaption to N− can be separated into three phases and eight functional clusters

**Fig. 1:**
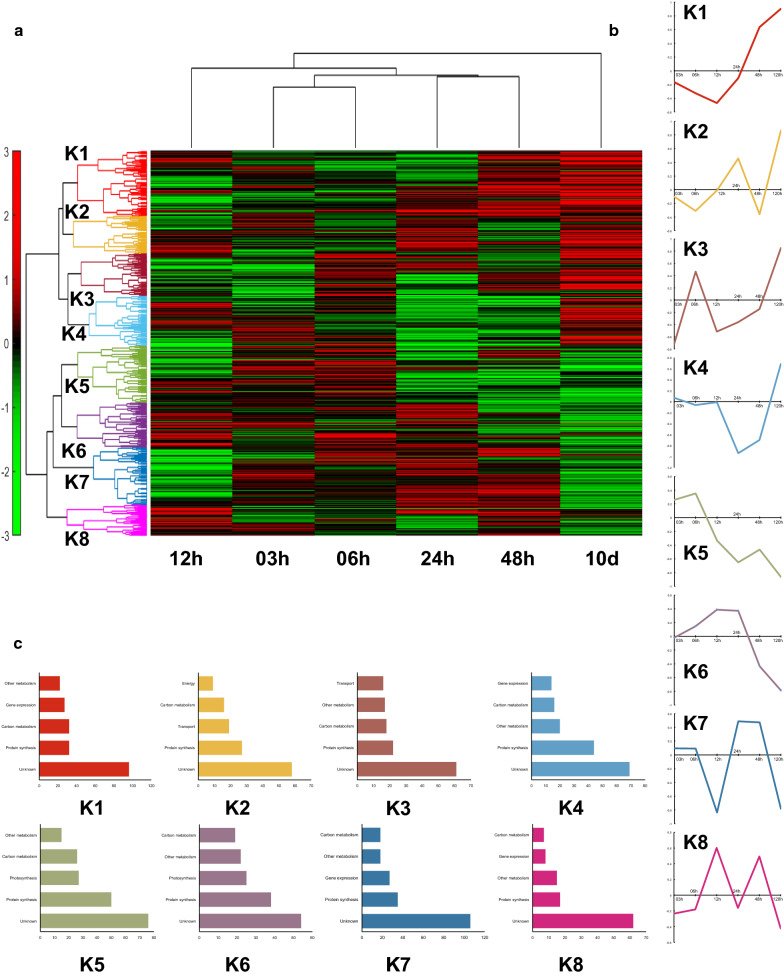
Global proteome profiling in response to N−/N+. **a** The hierarchal clustering of the protein fold changes log2LFQ (N−/N+). On columns are the six time points clustered based on Euclidean distances and on rows the fold change log2LFQ(N−/N+) of each protein clustered using Spearman’s collation. **b** The mean values of each row sub-cluster and 1C shows the top five enriched functions in each sub-cluster

To characterize the physiology upon switch from normal culture condition to N− in *N. oceanica* IMET1, proteomes were compared among culture samples from 3 h, 6 h, 12 h, 24 h, 48 h and 10 days, which provided proteome profiles of *N. oceanica* IMET1 cells under differing nitrogen availability. By employing label-free relative quantification (LFQ) for the proteome data, a total of 4114 protein sequences were identified and quantified. This represents about 41% of all theoretically predicted 9915 proteins encoded in the genome of *N. oceanica* IMET1 [6, 9, 35, 36]. After filtering out proteins identified under one treatment only in less than half of the samples, 1795 proteins remained. For these, hierarchal clustering was performed on the log2-fold change of label-free-quantified, normalized protein intensities (i.e., log2LFQ (N−/N+), as LogetP) [37, 38] (Fig. 1a). Functional category was based on homology from gene annotation data obtained by Blast P searches against non-redundant protein sequences (nr) database from NCBI (Fig. 1b, c) [31, 39]. On the time dimension, proteins were distributed into three phases over six timepoints, i.e., from 3 h to 12 h, from 12 h to 48 h, and until 10 days, with 12 h as distinct phase switch point. PCA analysis of protein LFQ-intensities of all samples also proved this separation from another angle (Additional file 1: Figure S1). In general, proteome profiles in the first 48 h were more similar, yet 12 h was an outlier and considered as physiology switching point as discussed later. Another significant distinction from other proteomes is obvious for 10th day (Additional file 1: Figure S1). This observation is partly mirrored by the clustering based on spearman’s correlation (Fig. 1a, b): on the level of changes in protein abundance, these 1795 proteins (N− vs N+) formed eight clusters with timepoints 12 h and 10th day displaying the strongest changes in individual clusters, e.g., clusters 7 and 8 exhibit strong up/down-regulation of proteins at 12 h and in most clusters 10th day exhibits the strongest up/down-regulation. To probe whether a given cluster was linked to specific protein functions, proteins were divided into twelve functional categories, and the five most frequent categories in each cluster (including genes of unknown function) designated as its primary functional proteins (Fig. 1b, c). Cluster K1 was functionally enriched with protein synthesis, carbon metabolism, gene expression, and other metabolism proteins, which were downregulated at 3 h and 6 h and then upregulated until 10th day (Additional file 2: Table S1). Although the primary functional genes of K4, K7 and K8 were all involved in carbon metabolism, gene expression, other metabolism, and protein synthesis, their tendencies were different (Fig. 1b): The proteins in K5 and K6, showing a downregulation trend, were related to protein synthesis, photosynthesis, carbon and other metabolism, but their tendencies were slightly different. K5 started with downregulation at 6 h, whereas in K6 proteins were upregulated first then downregulated at 24 h. In K2, a zigzag trend for protein ratios was displayed, energy (ATP)-related genes were enriched, as were proteins related to carbon metabolism, transport, and protein synthesis. In K3 proteins with functions in protein synthesis, transport, carbon metabolism, and energy were upregulated about one log2-fold change.

### On the level of functional categories, magnitude of changes was smaller for proteome compared to transcriptome

**Fig. 2:**
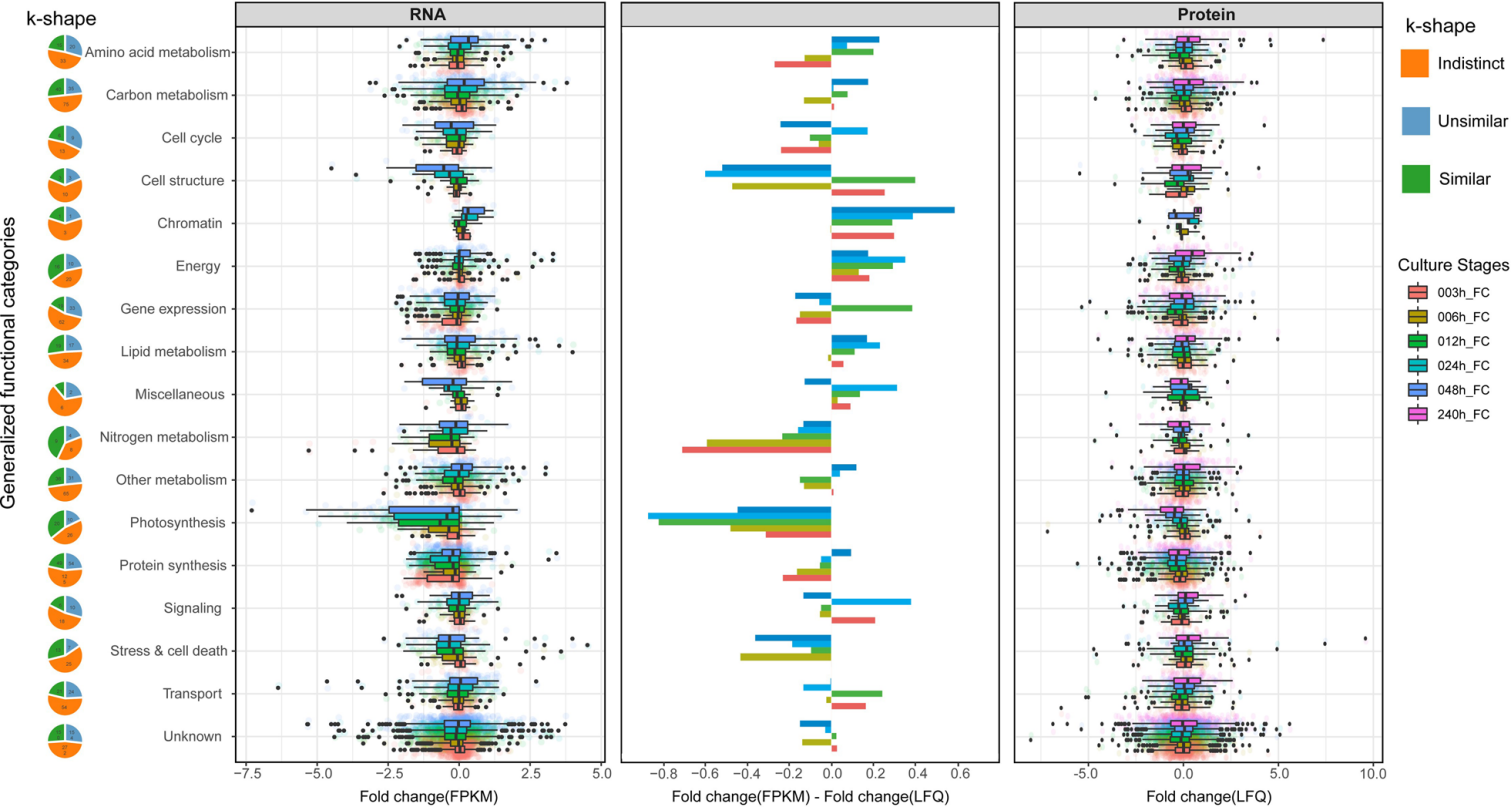
Comparison of N− with N+ condition for transcriptome and proteome in seventeen groups of functionally enriched genes. The first column shows the log2-fold change of transcriptome in FPKM (fragments per kilobase of transcript per million mapped reads) between N− and N+ obtained from Li´s data [6]. The third column shows the log2-fold change of proteome in LFQ (label-free quantification intensities) between N− and N+ . The middle column shows the difference between the transcriptome and proteome in log2FPKM(N−/N+)-log2LFQ(N−/N+), i.e., LogetT–LogetP since no transcriptome data of 10th day was published, the comparison was only between 3, 6, 12, 24 and 48 hrs

For the various functional categories, a corresponding previous time series of transcriptomes’ fold changes (retrieved from [6] including five time points 3 h, 6 h, 12 h, 24 h and 48 h) was contrasted with the proteome fold changes over the six timepoints of 3 h, 6 h, 12 h 24 h 48 h and 10th day (Fig. 2). Underlying ratios of proteins LogetP and the ratios of transcripts (log2FPKM(N−/N+), as LogetT) were listed in one table to facilitate further analysis (Additional file 3: Table S2). On the level of functional categories, temporal changes in proteome and transcriptome were mostly similar, yet some functional groups showed differences: at 12 h and 24 h transcripts of photosynthesis genes were more downregulated compared to their proteome counterpart. The strongest downregulation of transcripts of photosynthesis genes was at 12 h, whereas it was at 48 h for proteins. The chromatin group showed upregulation on transcriptome at 24 h and 48 h (factor around 0.5), whereas on the proteome, upregulation manifests at 10th day (Fig. 2 and Additional file 3: Table S2). In the nitrogen metabolism group, the downregulation for transcriptome is stronger than proteome. The proteins related to cell structure were downregulated more than their transcripts for 3 h and 12 h, whereas upregulated at 48 h (Fig. 2). In general, in most functionally enriched groups, the magnitude of changes for transcriptome was larger than for proteome, pointing towards a generally more stable proteome in *N. oceanica* facing N−.

**Fig. 3:**
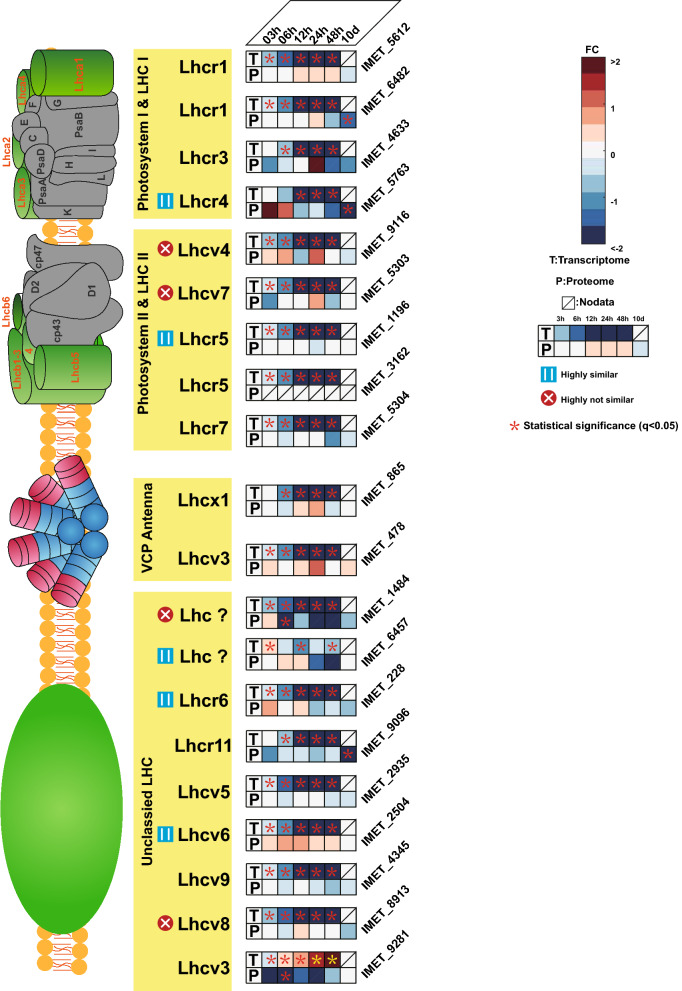
Fold change of transcriptome and proteome for light-harvesting genes. Heatmap shows the log2-fold changes (FC) of transcriptome in FPKM and proteome in LFQ intensity between N− and N+ . Based on their k-shape distances the similarity between transcriptome and proteome was denoted as highly similar, highly unsimilar and indistinct

**Fig. 4:**
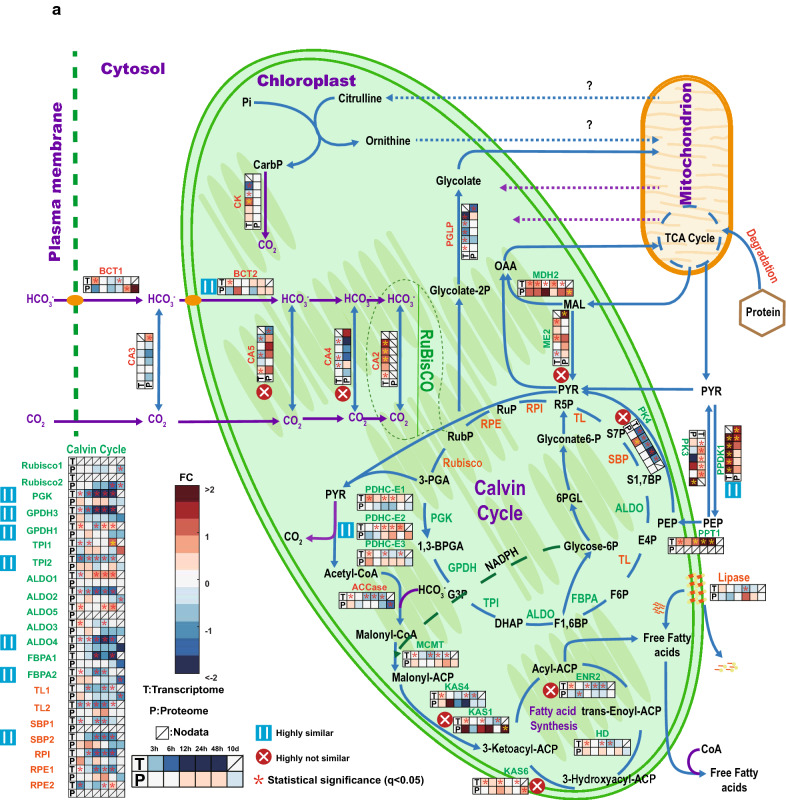

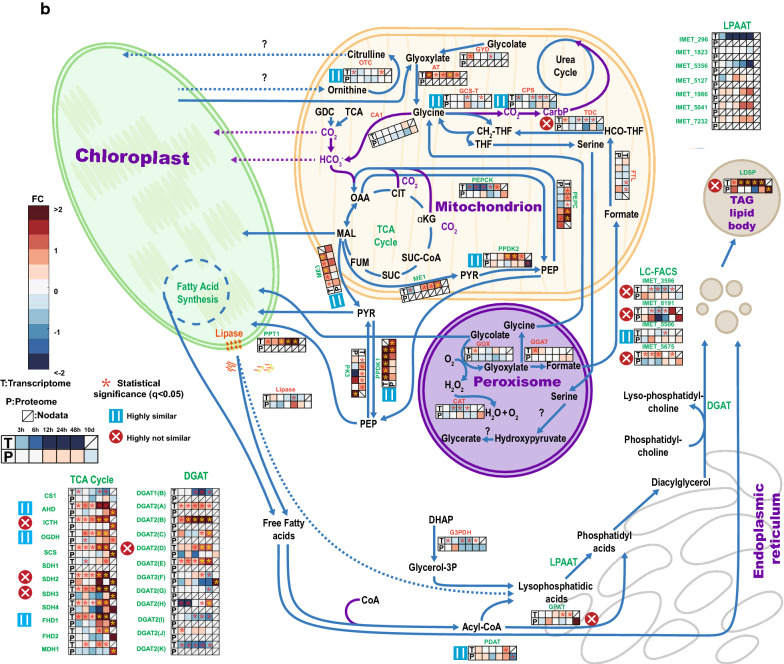
Overview of carbon fixation, transfer, and conversion to lipids. Heatmap shows the log2-fold changes of transcriptome in FPKM and proteome in LFQ intensity between N− and N+ . It presents the change tendencies of genes related to carbon fixation, transfer, and conversion to lipids. To compare our data to former data from Li et al. [6], those genes mentioned in both our and their study were labeled green and genes only mentioned in this paper were set to orange. Purple arrows show the carbon flows. **a** is about the photosynthetic pathways and **b** is about the reactions in mitochondrion and Kennedy pathway. Additional file 3: Table S2 contains the underlying data and full-length gene names

Given that mRNA synthesis precedes protein synthesis, it can be comprehended that the proteome response to changes in transcription may be delayed. To account for such delay, contrasting the tendencies of transcriptome and proteome rather than making a point-to-point comparison would be more appropriate [40]. For this purpose, Paparrizos and Gravano introduced a statistical method, so called “k-shape” [41]. Intriguingly, despite high point-to-point differences between protein and mRNA for photosynthesis genes in Fig. 2, according to k-shape only 18% of proteins exhibited tendencies highly unsimilar to their transcriptome data. Thus, for photosynthesis, majority of qualitative differences between protein and mRNA levels can be explained by a delayed proteome response to transcriptional changes (Figs. 3 and 4a).

### Transcriptomics overestimates downregulation of light-harvesting complex (LHC) proteins under N−

In the *N. oceanica* IMET1 genome, 20 genes are predicted as light-harvesting complex or VCP antenna proteins [42] of which nine proteins (IMET_6482, 4633, 5763, 1484, 6457, 228, 9096, 4345 and 9281) were downregulated on proteome and all except IMET_9281 downregulated on the transcriptome, respectively (Fig. 3). Moreover, based on the k-shape distance, five LHC proteins had change tendencies highly similar to their transcriptome (highly similar is defined as k-distance smaller than lower quartile of all the k-distances), which were also continuously downregulated. Four protein tendencies (IMET_9116, 5303, 1484 and 8913) were highly dissimilar to their transcripts (bigger than upper quartile of all the k-shape distances); the protein abundance of PSII Lhc4 (IMET_9116) was increasing under N− at 3, 6 and 24 h, and the PS II Lhc7 (IMET_9116) was increasing under N− at 48 h. The unclassified light-harvesting complex protein Lhcv8 was relatively stable over time. Generally, most LHC transcripts were considerably downregulated under N− from 3 h to 48 h except IMET_9281. However, corresponding proteins were relatively stable at beginning with onset of downregulation usually after 24 h (Fig. 3).

### Under N−, carbon fixation and transfer, as well as main carbon source for TAG synthesis change over time

*Nannochloropsis* under N− will accumulate lipids, mostly TAGs [6, 14]. The relevant processes of carbon fixation, transfer, and conversion to lipids were depicted both for transcriptome and proteome (Fig. 4a, b). Carbonic anhydrases (CAs) play a key role in biophysical carbon concentrating mechanisms (CCM) to catalyze the inter-conversion of CO_2_ and HCO_3_^−^ [36]. Five putative CAs (CA1: IMET_2109 CA2: IMET_5775; CA3: IMET_4525; CA4: IMET_1930, CA5: IMET_1034) are present in *N. oceanica* IMET1 [6, 25] and predicted to mainly reside in the chloroplast according to results from ChloroP, TargetP, Signal P and former research [41–46] (Additional file 4: Table S3). Under N−, on proteome level, CA1 and CA3 strongly increased in abundance at 10th day (log2LFQ(N−/N+) = 0.9 and 1.4), while CA2 increased during the first 48 h then decreased to -1 (LogetP at 10th day (Fig. 4a). In contrast, transcripts of the three CAs (CA1, CA2, CA3) were all slightly downregulated. It is noteworthy that, despite the transcript of CA4 was increasing over time, the corresponding protein was not detected. Regarding Bicarbonate transporters (BCTs) responsible for chloroplast HCO_3_^−^ transport, protein abundances increased at 48 h to 10th day (BCT1) and 6 h (BCT2), yet the transcript levels of these two bicarbonate transporters were quite stable (Fig. 4a).

**Fig. 5:**
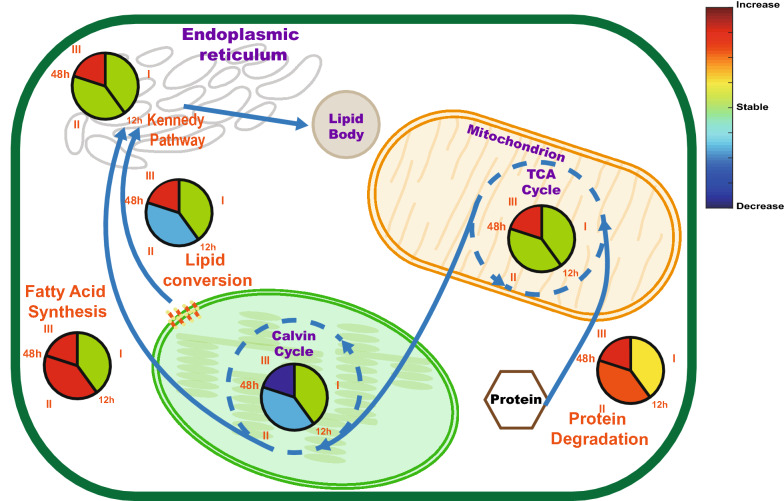
Main carbon sources for TAG synthesis and metabolic activities in different phases. The color-filled pie symbols show the regulation tendencies of pathways in different phases, the color was defined as the mean value of ratios of all the proteins in this pathway

The Calvin cycle plays a vital role in transforming the light energy into carbon compounds by fixing CO_2_ as glyceraldehyde 3-phosphate (GAP). On proteome level, the abundances of triosephosphate isomerase (TPI) and transketolase (TL) (IMET_71 and 7516) increased at 24 h and 10th day, whereas most of the other enzymes related to the Calvin cycle decreased under N− condition: The protein abundance of seven enzymes (IMET_8093, 9697, 1575, 3984, 4415, 5352 and 4727) started to decrease at 48 h with tendencies highly similar to their transcripts. For other enzymes of the Calvin cycle, like IMET_2880, 6907, 2540 and 4720, only their transcripts were identified. Generally, except TPI (IMET_71), which increased at 10th day, all the proteins related to the Calvin cycle were stable before 48 h of N− then started their down regulation. Unlike proteins, the transcripts of genes were reduced earlier and more (Fig. 4a) at 3 h, down to −3 and −2 LogetT after 24 h. Generally, unlike the transcripts indicating onset of reduced activity of Calvin cycle in Phase I (3 h–12 h), proteomics suggests that the decrease of the efficiency of Calvin cycle happened after 48 h of N− (Fig. 5).

The synthesis of TAG under N− is not yet sufficiently understood at the proteome level. In chloroplast exists the fatty acid synthesis pathway that catalyzes the synthesis of fatty acids from GAP obtained in Calvin cycle, [4, 45–49] (Fig. 4a). Alternatively, triosephosphates from Calvin cycle are used for biosynthesis of carbohydrates (Additional file 5: Figure S2). Therefore, both lipid- and carbohydrate-synthesis pathways are closely associated;

For carbohydrates, GAP is converted into glucose, and the reaction of glucose-1-phosphate to UDP-glucose is committed step for synthesis of cellulose and the storage carbohydrate laminarin. For the first 3 h, transcripts exhibited a slight increase for enzymes of glucose-1-phosphate synthesis, cellulose and laminarin. This was mirrored on the proteome level, though not significantly for glucose-phosphate mutase (IMET_2792, IMET_4273), UDP-glucose phosphorylase (IME_422, IMET_2792), cellulose synthase (IMET_4748, IMET_6523), except for laminarin branching enzyme (IMET_2418). Endoglycosidases were not detected on the proteome level – IMET_5082 mRNA was consistently upregulated, whereas IMET_7845 mRNA was downregulated at 24 h and 48 h.

For lipids, the GAP is converted to pyruvate, which is utilized for acetyl-coA synthesis by chloroplast pyruvate dehydrogenase (PDH, IMET_8724), which increased after 24 h both in transcript and protein amounts. Enzymes that catalyze conversion of malonyl-acp to 3-ketoacyl-acp were strongly upregulated (LogetP = 3) at 10th day under N−, with changes highly unsimilar to their transcripts. The protein abundance of ketoacyl synthase (KAS6, IMET_5417) under N− also increased at 10th day, but its transcription barely changed. In *Nannochloropsis*, the increasing abundance of fatty acid synthesis proteins on phase II (12 h-48 h) and phase III (10th day) indicated that after N− the photosynthesis switched from carbohydrate to fatty acid production to supply TAG after 48 h. On the other hand, the upregulation of lipase (IMET_5348) indicated the TAG synthesis until 24 h under N− may consume chloroplast lipids.

### The upregulation of enzymes on Kennedy pathway suggests that most of the TAGs were de novo synthesized after 48 h of N− depletion

The Kennedy pathway involves the synthesis of TAG from acyl-CoA. At the proteome level, only a minor portion of related enzymes were identified. Most of these proteins showed no strong changes (Additional file 3: Table S2 and Fig. 4b). The glyceraldehyde 3-phosphate dehydrogenase (GAPDH, IMET_ 3470) that provides glycerol-3-phosphate as backbone for fatty acid synthesis presented an increase at the protein level at 6 h. Glycerol-3-phosphate acyltransferase (GPAT, IMET_9161) was regulated highly differently at proteome level vs transcript level; its protein abundance showed a slight upregulation at 6 h, and strong increase up to 2 LogetP at 10th day, while its transcript remained unchanged. No lysophosphatidic acid acetyltransferase (LPAAT) was found at the proteome level, even though in the transcriptome seven isoforms were identified (Fig. 4b). Long-chain fatty acyl-CoA synthetase (LC-FACS) were reported to participate in lipid synthesis and transportation [50, 51]. Protein abundance changes of IMET_3596, 8191 and 5675 were highly unsimilar to their transcript changes. IMET_8191 was upregulated at 6 h and 10th day and IMET_5675 was slightly downregulated from 3 h, then upregulated at 10th day. For DGAT, only two proteins were identified. At the protein level, unlike its transcript, DGAT (IMET_1645) was downregulated at 3 h and 12 h and at the other time points not changed. The other detected DGAT protein was IMET_9521, from 3 h to 48 h, its protein abundance was downregulated, however, it was strongly upregulated at 10th day under N−. The lipid body surface protein (LDSP, IMET_5506) displayed similar trend for proteome and transcriptome; both hardly changed over time under N−.

### Mitochondrion contributed carbon precursors to the fatty acid synthesis via the protein degradation

The mitochondrion may possess a potential biochemical pathway for fixing inorganic carbon (HCO_3_^−^) postulated previously by us [25, 31]. The carbon was fixed by phosphoenolpyruvate carboxylase (PEPC, IMET_4839) converting phosphoenolpyruvate (PEP) to oxaloacetate (OAA) (Fig. 4b). From 24 h an increase in protein abundance began for PEPC (IMET_4839). It reached a LogetP of 1 3 at 48 h. Meanwhile, its transcript has been already upregulated since 3 h. In the TCA cycle, OAA is transformed to malate by malate dehydrogenase (MDH IMET_7304, 8706) to generate energy or transferred to chloroplast via C4 like pathway [6]. There was a strong upregulation on the protein level after 48 h for most OAA-related enzymes (MDH1: IMET_7304; pyruvate phosphate dikinase (PPDK2: IMET_5132; PEPC: IMET_4839, and phosphoenolpyruvate carboxykinase (PEPCK, IMET_6484), while their transcripts increased after 24 h of N−. For the other enzymes related to the TCA cycle, all proteins except citrate synthase (CS1, IMET_1911) were strongly upregulated at 10th day, succinate dehydrogenase (SDH, IMET_1564) was upregulated up to factor 3.6 LogetP, transcripts of genes IMET_706, 9373, 7535, 1564 and 1900 have been strongly upregulated from 24 h of N− (Fig. 4b).

In cytoplasm, the enzyme catalyzing conversion of pyruvate (PYR) to PEP (IMET_3227) strongly increased from 24 h to 10th day and has been at transcriptome level upregulated from 3 h of N−. On the contrary, the enzyme catalyzing conversion of PEP to PYR, pyruvate kinase (PK, IMET_8583), was strongly downregulated at 24 h and 10th day on transcription level with no big changes in protein abundances. The other genes involved in the so called C4-like pathway, such as IMET_3280, were also upregulated, though here protein abundances increased later than that of transcripts. In summary, the upregulation of TCA cycle enzymes points to increasing MAL and PYR concentrations from 48 h, which provided the carbon precursors for fatty acid synthesis (Fig. 5).

### Nitrogen metabolism differs in L-glutamate synthesis between proteome and transcriptome

**Fig. 6:**
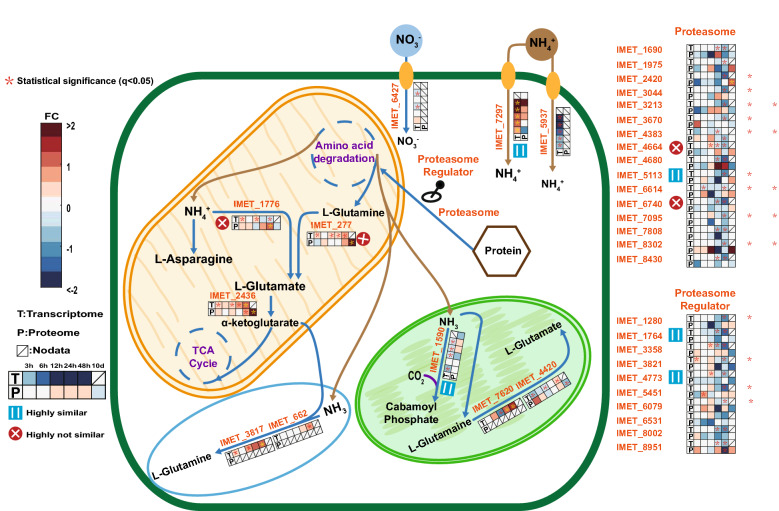
Key processes of nitrogen turnover. Heatmap shows the log2-fold changes of transcriptome in FPKM and proteome in LFQ intensity between N− and N+ with predicted localization of the proteins

Several proteins involved in nitrogen transport and metabolism were not detected, but glutamate dehydrogenase (IMET_1767) and glutamate synthase (IMET_277). Glutamate dehydrogenase catalyzes the synthesis of L-glutamate from ammonia and alpha-ketoglutarate, whereas glutamate synthase converts l-glutamine and alpha-ketoglutarate into two molecules L-glutamate and increased at the protein level. Both enzymes IMET_1767 and IMET_277 were identified with dissimilar changes at the protein and transcript levels (Fig. 6) from 12 h to 48 h. Ammonium importer (IMET_7297) was strongly upregulated at 48 h at the protein level. Nitrate reductase (IMET_1590) hardly changed at both protein and transcript level. Proteasome is the first step of protein degradation, in Fig. 6, the proteasome subunits (IMET_1975, 3213 and 8302) were upregulated at 12 h. Later, at 10th day, more proteasome subunits like IMET_1975, 2420, 4664, 5113, 6614 and 8302 were upregulated. On the contrary, the transcriptome of proteasome was stable or slightly downregulated. Proteasome regulators are considered as activities regulator of proteasome. At the protein level, most proteasome regulators (IMET_1280, 1764, 3358, 3821, 4773, 5451, 6079, 6531 and 8002) were stable until 48 h and downregulated at 10th day, except the IMET_8951, which was upregulated at 3 h and 10th day.

## Discussion

### Temporal adaptation of *Nannochloropsis* to N− stress occurs in distinct phases

Based on cluster and PCA analysis of proteomes (Fig. 1a and Additional file 1: Figure S1), the period of *Nannochloropsis* N− to the tenth day was divided into three phases, i.e., 3 h to 12 h, 12–48 h, and 10 days, where 12 h played a switch point and day 10 described long term adaptation. Owing to the proteome composition at 12 h being distant from 3 to 6 h and 24–48 h, we speculate that here cells start switching from an overall N+ to an overall N− physiology characterized by largely increased TAG accumulation and chloroplast reorganization. Before 12 h, the cells were in the transition stage of adapting to the N− environment, and some lipids began to synthesize [6, 28]. Since the chloroplast morphology has not changed significantly [14] (Additional file 6: Figure S3) in 12 h, it was speculated that the lipid may be derived from protein decomposition [6, 14] and photosynthesis [52, 53], though the latter probably contributed most according to a study with *N. gaditana* [54]. The second phase of 12–48 h was beginning of re-organization of cellular structure and physiology as corroborated by other studies: the size of the chloroplast still was not visibly changing, but the total cellular proteins declined, while the lipid content was rising [14] and the diversity of TAG molecular increased after 12 h [6]. Massive morphological re-organization occurred from 48 h to 10 days: changed chloroplast size became visible between 4th and 6th day (Additional file 6: Figure S3). Until the 10th day, the chloroplast reversibly continued to shrink [14] and displayed residual photosynthetic carbon fixation [20], and the size of the lipid body in the N− cells continued to increase to maximum [14].

Different phases of adaptation were found for carbon-related metabolic pathways, too. Generally, the transcripts of genes located on the fatty acid synthesis pathway in chloroplast slightly changed expression after N− and the enzymes on the Kennedy pathways were downregulated at the earlier periods of N− and started to be upregulated after 24 h of N−. During the first phase from 3 h to 12 h, the transcripts and proteins of most genes related to photosynthesis, carbon metabolism and energy metabolism were stable, except the upregulated enzymes (beta-ketoacyl-acyl-carrier-protein synthase ii: IMET_1043 and IMET_3842) which catalyze the malonyl-ACP to 3-ketoacyl-ACP conversion and the upregulated enzymes in chloroplast that catalyze reactions of MAL to OAA or to PYR (Fig. 4a). These may indicate that the de novo synthesized lipid after 24 h of N− is mainly from the fatty acid synthesis pathway [6]. The second phase is from 12 h to 48 h, the transcripts of Calvin cycle-related genes started strong downregulation, although their protein amounts remained unchanged. On the contrary, concurrently the transcripts of genes that participate in TCA cycle were strongly upregulated, and their protein became upregulated at 48 h. At this phase of N-starvation, the *Nannochloropsis* cells started to accumulate a variety of TAGs [6]. The enhanced flux of TCA cycle may provide carbon precursors that TAG synthesis required [14, 55]. During phase III, i.e. 10th day, the proteins of Calvin cycle were strongly downregulated and the proteins of Kennedy pathway and TCA cycle were strongly upregulated to reach maximal TAG amount [14].

### The change tendencies of LHC proteins and upregulated CCM on the protein level suggest continuous carbon supply from photosynthesis for TAG synthesis

Photosynthesis was detected as one of the carbon resources for fatty acid biosynthesis after N− [50–54]. Generally, N− had a big impact on the chloroplast composition: not only the chlorophyll, but also the carotenoids decreased [54–58] and decline of chloroplast lipid content occurred [14]. The photosynthetic electron flow was rebalanced in N− cells, indicating that the decrease in *Fv/Fm* values in N− cells may be part of the adaptation process to N− to relocate the nitrogen to the essential cellular metabolism process [57, 59]. Intriguingly, previous research on the transcriptome suggested that all the LHCs were downregulated, including both PSI and PSII systems [53, 54, 58]. However, our protein data indicated that LHCs belonging to the PSII system were less affected by N− than those of PSI (Fig. 3). Importantly, former research on *Nannochloropsis gaditana* has pointed out that decrease of activity of PSII is bigger than that of PSI in N-starved cells. The PSII proteins still existed in the N− cells, but the number of active PSII reaction centers were strongly decreased [60], smaller activity can be explained by lower turnover rate of D1 subunits of PSII system and migration of LHCs from PSII towards PSI [57, 61]. The D1 subunit must undergo a fast turnover in the light due to photodamage, its rate is limited by the speed of protein degradation [62]. Our protein data suggested that the protein abundance of PSII D1 was only slightly downregulated under N− (Additional file 3: Table S2) while at the same time the activity of PSII was strongly downregulated [57], which indicated damaged D1 protein underwent slower degradation. Therefore, we postulate that reduced D1 turnover rate resulted in less active of PSII. Generally, the combined transcriptome and proteome data corroborated the speculation from former research that decreasing photosynthesis efficiency was mostly due to the loss of PSII activity and was also compatible with the hypothesis that LHC proteins from PSII migrate toward PSI [57, 61].

Figure 4a shows a model of a possible biophysical CCM transport path in *N. oceanica* Imet1 under N−/N+. Two CCM transport routes are shown [63, 64]. On one side is the direct diffusion of dissolved carbon dioxide into and out of the cell, on the other side is the active transport of bicarbonate via bicarbonate transporters (BCT, IMET_18 and IMET_1774), which was enhanced at 48 h to 10th day. As described in results, the transcriptome and proteome of CA enzymes had opposite trend (IMET_5775 and 4525). The increase of CA and BCT enzymes on the protein level support the idea that after 48 h and longer N− carbon was still fixed by facilitating transfer of inorganic carbon to Rubisco to participate in the Calvin cycle [52]. Although the transcriptome of most Calvin cycle-related genes started to down regulate at 24 h of N− deprivation (Fig. 4a), their proteins remained stable. At 10th day, the enzymes (Rubisco, PGK, and GPDH) catalyzing RuBP conversion to GAP were only slightly downregulated and the TPI (IMET_71) even strongly upregulated. These indicated that even after 10 days of N−, the pathway used to fix carbon by Rubisco was still functioning. This proteome-based hypothesis is supported by several other publications [65, 66]. Hence, ongoing carbon fixation may supply the fatty acid synthesis pathway inside chloroplast (Fig. 4a).

These findings have implications for strain improvement and demonstrate that engineering of photosynthesis is more relevant than previously suggested. The results suggest to further explore means to tailor photosynthesis guided by findings for other organisms, e.g., improvement of light quantum yield by reducing antenna size [67], increasing NADPH supply for FA synthesis [68]. Furthermore, ACCase and MCMT, catalyzing the first steps of FA synthesis in chloroplast did not increase or even decrease even after 48 h. Interestingly, overexpression of MCMT increases total lipid production in *N. oceanica* [69] and ACCase in *P. tricornutum* [70]. Therefore, overexpression of ACCase may enhance lipid production in *N. oceanica*, too.

### Mitochondria play important role in TAG synthesis by membrane lipid degradation and FA precursors production

The previous research on vascular plants suggested that mitochondria play an important role in TAG synthesis by providing fatty acid precursors for the FA elongation in the cytosol and de novo FA biosynthesis in the plastid [69–73]. Moreover, because in vascular plants the β-oxidation of FA occurs in mitochondria and peroxisomes, it was speculated that mitochondria may contribute to the *Nannochloropsis* TAG synthesis under N− by recycling carbon skeletons from degraded membrane glycerolipids [20]. The galactolipases are known in vascular plats as the key enzymes that hydrolyze galactolipids with the release of fatty acid and show in particular activity under stress [74, 75]. Therefore, the patatin enzyme (IMET_3177) with predicted mitochondrial localization may play an important role in membrane lipid degradation (Additional file 4: Table S3) [76]. Although its expression barely changed according to transcriptomics, its protein abundance was upregulated 2 times at first 12 h (Additional file 4: Table S3). This could be an indication of an early activity of this lipase and potential evidence that membrane lipid recycling may have also occurred in early phase of N−. At later time points (24 h and 48 h) its enzyme abundance displayed no difference between N− and N+ cells, and even decreased at 10th day. Thus, in the later phase (after 6th day) of N-starvation the membrane lipid recycling should not be the main TAG synthesis resource; this might also in part be due to decreased synthesis of chloroplast lipids indicated by decrease in MGDG synthase (IMET_4672) at 10th day (Additional file 3: Table S2). As it can be seen in Additional file 4: Table S3, the abundance of a lysophospholipase (IMET_257) was slightly upregulated at 3 h, and from 24 h stronger (> 1, LogetP) upregulation of this protein took place. This may directly translate to higher activity of this phospholipase after 24 h. Therefore, this phospholipase (IMET_257) is a potential candidate to contribute to lipid membrane recycling.

Protein degradation can contribute carbon precursors for TAG synthesis and nitrogen to maintain the essential metabolic processes under N−. In the ubiquitination-meditated N-degron pathways of protein degradation, proteasome plays an important role [77]. As described in result part, the upregulation of proteasome subunits occurred for some at 12 h and even more at 10th day, which indicated the degradation of protein has two phases: the quick reaction at 12 h and phase III (10th day). On the other hand, the proteasome regulators, that can enhance or inhibit the activities of proteasome, were stable and started to decrease at 48 h and phase III. The proteasome activator IMET_8951 is the only exception, it was upregulated at 3 h, 12 h and 10th day which indicated the upregulation of proteasomal activity at these phases. Generally, glucogenic amino acid backbones are first directed to the TCA cycle, from where further transformation to PEP may occur. Ketogenic amino acids are transformed into acetyl-CoA and acetoacetyl-CoA. Hence proteinogenic carbon could be directly or indirectly directed towards TAG synthesis. Some proteinogenic carbon may be utilized via PEP in gluconeogenesis for glucose synthesis. This is in line with publication [14], describing that total protein amount decreased after N−, whereas glucose content increased. These previous findings and the upregulation of PK and PPDK (IMET_3227 and 8583) (Fig. 4a) on the protein level indicated the degradation of protein may be an important carbon resource for the TAG synthesis and redistribute nitrogen after N−. The latter process was further suggested by the strongly increased abundance of enzymes catalyzing conversion of glutamate and glutamine (IMET_2436, IMET_277) at 48 h and 10 days, which may indicate the use of N-recycling to synthesize amino acids. It is noteworthy that protein level of PK and PPDK enzymes began to increase after 24 h of the N−. Hence, there is reason to speculate that the response of *Nannochloropsis* to N− is staged: the carbon source required to TAG synthesis was early from the membrane recycling and photosynthesis and at later stage additionally from protein degradation.

After 24 h of N−, the transcripts of all enzymes in the TCA cycles except citrate synthase were upregulated (> 2 LogetT) (Fig. 4b). At the same time, the transcript of anaplerotic PEPC synthesizing OAA from PEP (IMET_4839) was also significantly upregulated. The upregulation on the protein level was delayed and started at 48 h up to 10th day. The upregulated OAA synthesis enzyme corroborates the hypothesis of TCA cycle as carbon supply for lipid biosynthesis [23, 78].

Of note, the most significant increase (> 4 LogetT) in the transcripts among these TCA genes was found for fumarate hydratase (IMET_706) synthesizing malate. Our proteomics analysis reveals that unlike the transcriptome indicated, the upregulation of TCA-related proteins mostly happened after the first 24 h of N− treatment. This includes IMET_706 (AHD). Malate can be subsequently exported to the cytosol or into the chloroplast via so called “C4- pathways” for the production of pyruvate by a NAD(P)-malic enzyme (ME). There were three MEs identified inside *Nannochloropsis* cells and predicted to be localized in the peroxisome/mitochondrion (IMET_2150), in the plastid (IMET_3280), and in the cytoplasm (IMET_7778) (Fig. 4). The transcript and protein of IMET_3280 were both upregulated. Therefore, unlike the speculation made by Li et al. [6], we have accumulated protein evidences to propound existence of the C4-like pathway in *Nannochloropsis*. The C4-like pathway would redirect carbon dioxide to the plastid and provide part of the pyruvate required for FA synthesis.

### Improved temporal model of TAG synthesis in *N. oceanica* IMET1 under N−

As described in Results, most of the enzymes on Kennedy pathway that synthesize TAG were upregulated under N-deprivation. Although the direct metabolic evidence about how much lipid was contributed by this pathway was lacking, published isotope labeling experiment indicated part of de novo synthesized lipid was from carbon fixed by Calvin cycle [52]. Janssen et al. proved most fatty acids accumulating in neutral lipids (NL) are synthesized de novo during N− [79]. Preexisting fatty acid (e.g., palmitic acid, palmitoleic acid) in the polar lipids (PL) were degraded but not translocated to the neutral lipids during N−. In contrast, preexisting eicosapentaenoic acid (EPA) in the PL was both degraded and translocated into NL at comparable rates, i.e., the turnover of chloroplast membrane lipids contributed a small part to the accumulated TAG [80]. Both processes, lipid recycling from the chloroplast membrane and the de novo synthesis of TAG under N− have been described for *N. gaditana* [57]. Fatty acid synthesis pathway creates fatty acid from acetyl-CoA and NADPH. This process takes place in the chloroplast of algal cell. As described above, for *Nannochloropsis* cells in the N−, the increase of carbohydrates and TAG started at 48 h [14]. Based on the increase in proteins with function of protein degradation (Fig. 5) and reported decrease in cellular protein [81], we speculate that the fatty acid synthesis pathway for TAG is supplied with acetyl-CoA from ketogenic amino acids as result of protein degradation and is significantly upregulated from 48 h, which can partly explain why TAG diversity increased significantly after 24 h [6, 34, 57].

Our data corroborates the observation of early increase in carbohydrate synthesis for *Nannochloropsis* [14], manifested as cellulose enrichment in the cell wall [82]. However, it has also been reported that the total and storage carbohydrate content is quite stable or increases over time [16]. In *Chlamydomonas* it has been proven that reduction of starch synthesis will increase the TAG accumulation [83]. In contrast, knockout of β-1,3-glucan synthase gene in *P. tricornutum* impaired growth and photosynthetic capacity and increased not only lipid content, but soluble sugars, too [84].

It has been revealed that under N-starvation, genes encoding pyruvate dehydrogenase complex (PDHC), and related genes are substantially upregulated [6]. Consequently, the acyl-CoA to synthesize TAG may also be derived from pyruvate via protein degradation. For enzymes on the Kennedy pathway like GPAT (IMET_9161), DGAT-2D (IMET_9521), their upregulation was observed at 10th day. In addition, LC-FACS associated with TAG synthesis and transport, and the LDSP (IMET_4666) on the lipid body also were strongly upregulated at 10th day, which matches with strongly increasing TAG amount after 48 h [6, 14, 34]. Finally, it should be mentioned that several important enzymes for this process like LPAAT and most DGAT were not detected with proteomics. This may be due to the physicochemical properties of membrane proteins and their relatively low abundance.

### Comparison of transcriptome and proteome using k-shape approach

To investigate the interactions between transcriptomes and proteomes, the common approach is point-to-point comparative analysis: i.e., the transcriptome is compared to the proteome at the same time point [19, 85]. However, Abraham’s paper revealed a temporal delay in the expression of proteins related to transcription, and proposed a “shift” method to investigate the relationship between them [40]. Here we introduce the mass centroid based k-shape method to study the relationships between proteome and transcriptome [41] for *Nannochloropsis* under N−. Compared to the “shift” method, k-shape method can better adapt the different delay times of the protein expression and has a better accuracy than all scalable approaches [41]. This method confirmed for several proteins delay in abundance changes of proteome vs. transcriptome as likely explanation for point-to-point differences; for example IMET_706, though differing in transcript and protein changes according point-to-point comparison was considered highly similar by k-shape, because changes at protein level occurred 24 h later than at transcript (Fig. 4a). This observation is of consequence for mechanistic interpretation in at least two aspects (a) protein abundance inference based on transcript data and (b) disclosure of regulation levels. Regarding protein abundance inference, this work recommends conducting time-series experiments in transcriptomics. In this vein, it is advised and feasible to consider adjacent time points for inference of protein abundance changes. Nevertheless, in case of more intricate transcript/protein regulation mechanisms, even considering temporal shifts is insufficient for the inference of protein from mRNA. As a matter of fact, many such cases were unveiled by applying k-shape in this study and were denoted “highly dissimilar” protein vs. transcript regulation trend. The k-shape method to realize the existence and extent of regulatory mechanisms working on post-transcriptional level could be exploited as aid in metabolic engineering endeavors. For instance, it would instruct how likely an increase in enzyme abundance can be achieved by common gene overexpression approaches.

## Conclusion

Proteomics over the time course of N− and its comparison to the transcriptome provided a framework for better understanding the metabolic changes of the oleaginous microalga *N. oceanica* IMET1. Compared to the reported temporal changes of transcriptome [6], the proteome exhibited distinct adaptation phases, delayed response to transcription and sometimes completely opposite regulatory trends. Proteome data for LHC, CCM, and the C4-like pathway underpin previous findings that photosynthesis and carbon fixation are still ongoing even after 48 h of N−. This conclusion is contrary to our previous transcriptome-based speculation [6], but more consistent with the results of the isotope labeling experiment which revealed that most lipids are de novo synthesized and originate from photosynthesis [52, 79]. Proteomics for TCA cycle and lipid metabolism indicated that these pathways in *Nannochloropsis* could roughly be divided into temporal phases of adaptation to N−, i.e., the “phase I” (3 h–12 h) of lipid recycling and the “phase II and III” (12 h–48 h and 10th day). Previously reported [14] lipid recycling and degradation of proteins for de novo fatty acid synthesis in phase II and III was substantiated. From the perspective of TAG synthesis, proteomics underpins metabolic analysis that found TAG de novo synthesized from acetyl-CoA and acyl-CoA via the fatty acid synthesis pathway and Kennedy pathway are the majority, and the TAG from PL recycling contributes only little to accumulated TAG [80]. General increase in abundance of key enzymes for protein degradation and nitrogen turnover in phase III explain how amino acid degradation may provide the nitrogen to sustain essential cell metabolic processes [14, 57].

## Materials and methods

### General experimental procedures of N−

*Nannochloropsis oceanica* IMET1 was inoculated in the modified f/2 liquid medium [6]. The cells were grown in 1-L glass tubes at 25 °C under continuous light (approximately 80 ± 5 µmol photons m^−2^ s^−1^) and aerated by bubbling with compressed air. After cell number measurement (OD_750_) and axenic checks [86] (Additional file 7: Figure S4). Mid-exponential phase microalgal cells (OD_750_ of 5.33 ± 0.35) were collected and washed twice with axenic modified f/2 medium without sodium nitrate (N− f/2 medium). Three tubes with fresh modified f/2 liquid medium and the other three tubes with N− fresh modified f/2 liquid medium were re-inoculated. Then, at the time point of 3 h, 6 h 12 h, 24 h, 48 h and 10th day, 100 ml samples were taken for protein analysis and OD_750_ measurement. At 10th day, the axenicity of cultures was checked again with the same method as before. Proteins were extracted from time point samples.

### Culture conditions of the *N. oceanica* IMET1 strain

The cultivation of *N. oceanica* IMET1 was optimized and modified according to published work [6]. The cultivation of *N. oceanica* IMET1 was performed in three steps. In the first step, cells were suspended in 100 μL f/2 medium (35 g L^−1^ sea salt (Real Ocean, USA), 1 g L^−1^ NaNO_3_, 67 mg L^−1^ NaH_2_PO_4_*H_2_O, 3.65 mg L^−1^ FeCl_3_*6H_2_O, 4.37 mg L^−1^ Na_2_EDTA*2H_2_O, trace metal mix (0.0196 mg L^−1^ CuSO_4_*5H_2_O, 0.0126 mg L^−1^ NaMoO_4_*2H_2_O, 0.044 mg L^−1^ ZnSO_4_*7H_2_O, 0.01 mg L^−1^ CoCl_2_, and 0.36 mg L^−1^ MnCl_2_*4H_2_O), and vitamin mix (2.5 µg L^−1^ VB_12_, 2.5 µg L^−1^ biotin, and 0.5 µg L^−1^ thiamine HCl)) from a glycerol storage culture (under sterile conditions) and spread on an agar plate (modified f/2 medium with 1.5% agar). This plate was cultured at 8 µmol photons m^−2^ s^−1^, 25 °C without CO_2_ fumigation in a programmable growth chamber (Model SWGC-450, Witeg, Germany) until individual colonies formed. A single colony was removed from this plate and suspended in about 50 ml of f/2 medium (modified according to [87]) and in a shaking flask for a further 5 days (at 50 µmol photons m^−2^ s^−1^ 25° C without CO_2_ fumigation) to an OD_750_ of 5. The culture was pelleted and resuspended in fresh f2 medium in a 1-L glass tube. The cultivation in the algae tube was carried out under the same conditions as in the growth chamber but with aeration by sterile air. The growth of the culture was monitored photometrically at OD_750_.

After start with an OD_750_ of 1.0, six cultures were prepared with modified f/2 medium for 7 days. Subsequently, the cells were pelleted and washed twice in f/2 medium without the nitrogen source (NaNO_3_). The cell pellets were transferred to the new sterile glass tube, resuspended and cultured for a further 10 days under N−. Three biological replicates of algal cultures, corresponding to altogether six column reactors, were cultivated under the N− and N+ conditions, respectively. Cell aliquots were taken at 3, 6, 12, 24, 48, and 10 days from each column by pipette for OD_750_ measurement and proteomic profiling. Bacterial contamination was tested after DAPI staining under a fluorescence microscope [86] at 0 h and 10th day.

### Protein extraction, preparation

*Nannochloropsis* cells were collected by centrifugation at 4 °C (Beckman Coulter Allegra X-12R, SX4750 Rotor, 2000 g, 10 min), then the cell pellets were freeze dried, weighted and ground seven times with liquid nitrogen. A tablet phosphatase inhibitor including protease inhibitor cocktails (Phos STOP, Roche) was dissolved in 1 mL of ddH_2_O. The inhibitor solution was mixed in a ratio of 1:9 with an SDS lysis buffer (4% SDS in 0.1 M Tris/HCl pH 7–8). The fragmented cells were mixed in the ratio 1:20 (w/v) with SDS lysis buffer upon thorough mixing and subsequent incubation at 70 °C for 4 min, the sample was centrifuged at 4 °C (Eppendorf 5415R, rotor: F54-24-11, 12,000 rpm, 20 min). The supernatant contained the extracted proteins. and was stored on ice. The concentration of the protein was determined by the BioRad^®^ DC assay kit with a BSA calibration line consisting of increasing concentrations of BSA in SDS lysis buffer.

One-dimensional 12.5% (v/v) polyacrylamide gel electrophoresis served for purification and solubilization of proteins. The separating gel was poured and covered with isopropanol for a straight gel front. The separating gel was polymerized for about 2 h. Isopropanol was removed and the stacking gel was poured over the solid separating gel. After a comb for the gel pockets was inserted, the stacking gel was polymerized after another 2 h. 50 µg protein sample was increased by using SDS loading buffer (3% w/v) loaded into one of each gel pockets, then the gel was run at room temperature, 300 V and 30 mA until bottom.

### Gel staining and discoloration

Coomassie “Silver Blue stain” was used for protein visualization (Candiano, Bruschi et al., 2004). The gel was washed twice with distilled water for 10 min to remove the SDS. Then the gel was dyed with Silver Blue stain solution overnight and washed twice with ddH_2_O after staining. Protein bands were excised from the stained gel, cut into small cubes (ca. 1 × 1 mm^3^) and distained in 150 μl distaining solution (10% ethanol and 2% phosphoric acid) for 30 min at 37 °C and 550 rpm in Thermomixer (Comfort, Eppendorf). This procedure was repeated two to three times until the gel pieces and de-staining solution no longer displayed a blue color. Thereupon, de-staining solution was removed, and the gel pieces were dried at 30 °C in a Speed Vac.

### In gel digestion

The dried gel pieces were completely immersed in digestion solution (~ 200 µl). The digestion solution consisted of sequencing grade modified porcine trypsin (Promega, Madison, USA), which was diluted in 40 mM ammonium bicarbonate (pH 8.6) to a concentration of 12.5 ng µl^−1^. The protein digestion was performed over night at 37 °C with tempered shaker (HLC MHR20, 550 rpm). After protein digestion, the peptides were extracted from the gel pieces as supernatant and were transferred to autosampler vials (12 × 32 mm^2^ glass screw neck vial, Waters, USA). The extracted peptides were dried using a Speed Vac at room temperature and stored at RT.

### LC–ESI-MS/MS analysis

For MS analysis of the trypsin-digested proteins, dried peptides were resuspended in buffer A (0.1% formic acid (FA) in HPLC class water (Fischer Scientific, GmbH (Germany)) and sonicated for 10 min in an ultrasonic bath (RK-100 H, Heidolph). The LC–ESI-MS/MS system consisted of a nanoACQUITY gradient UPLC pump (Waters Corporation, USA) interfaced to an LTQ Orbitrap Elite mass spectrometer (Thermo Fisher Scientific, USA). For LC, an ACQUITY UPLC 2D VM M-Class Symmetry C18 trap column (100Å, 5 μm, 180 μm x 20 mm) (Waters Corporation, USA) was coupled to an HSS T3 ACQUITY UPLC M-Class separation column (75 μm × 150 mm) (Waters Corporation, USA). The nanospray source was a PicoTip Emitter Silica Tip (10 μm tip ± 1 μm) (New Objective, USA). Xcalibur (Version 2.2 SP1) was used for the software-based instrument control of the mass spectrometer.

For the UPLC method, the flow rate was 0.4 μL/min. A 105 min gradient was used with 0–5 min: 2% buffer B (0.1% formic acid in acetonitrile, UPLC/MS, Fischer Scientific, GmbH (Germany)); 5–10 min: 2–5% buffer B; 10–71 min: 5–30% buffer B; 72–77 min: 85% buffer B; 77–105 min: 2% buffer B. The analytical column oven was set to 55 °C and the heated desolvation capillary was set to 275 °C.

For the MS analysis, a full scan was first performed in the Orbitrap in the range of 150–2000 *m/z*, then the 20 most intensive precursor ions from the full scan were fragmented using the CID method (activation time 10 ms and 35% collision energy). The resulting fragments were detected in the ion trap. All precursors of unknown charge or charge ≠ 2 or 3 were rejected for MS/MS analysis.

### Protein identification and label-free quantification

Max Quant (Version 1551) with Andromeda search engine was used for protein identification and label-free quantification (LFQ) [37, 88, 89]. The protein identification was against the complete proteome database of *N. oceanica* IMET1 [9]. The mass tolerance for calibrated precursor ions was set to 4.5 ppm; the mass tolerance for fragment ions was set to 0.6 Da. Only tryptic peptides with up to two missed cleavages were accepted. The oxidation of methionine, acetylation on protein N-terminus and propionamide on cysteine were admitted as a variable peptide modification. The false discovery rate (FDR) was 0.01 for protein. For protein quantification, the “Label free quantification (Max LFQ)” function in MaxQuant was used [89]. This method is based on the “delayed normalization” strategy, which after the identification of peptides determines the normalization coefficients for each LC–MS/MS run.

For the comparison between N− and N+ proteomes, the LFQ-normalized intensities of samples, which were generated by MaxQuant, were transformed to log2 with the software Perseus [90]. For sample comparison, proteins not quantified in at least half of the time points for N− and N+ were removed. For the remaining 1795 proteins missing values were imputed using imputation from normal distribution in Perseus (width 0.3, down shift 1.8). The fold changes between N− and N+ samples were compared (i.e., log2 LFQ (N−/N+)) with two sample *t* test (FDR = 0.05). All the other analyses were carried out in the MATLAB^®^ (2017, The MathWorks) and R^®^ (3.6.1, R Core Team) environment for statistical computing and graphics.

### Predicting the subcellular localization of proteins

A series of online software tools were used to predict protein subcellular localization. According to the results, the possible compartmentalization of CCMs, central carbon metabolism, photorespiration metabolism in *Nannochloropsis oceanica* IMET1 was defined. Firstly, SignalP was used to predict the secretory signal peptide that targets its passenger protein for translocation across the endoplasmic reticulum membrane in eukaryotes [91]. Secondly, ChloroP, which presents a neural network based method for identifying chloroplast transit peptides and their cleavage sites [43], was employed. Thirdly, the program TargetP was employed to evaluate mitochondrial, chloroplastic, secretory pathway or other subcellular location [45, 46]. Results from these programs were combined and those with majority consensus were chosen as the predicted localization for a particular protein (Additional file 4: Table S3).

### Transcriptome data preparation

The transcriptome data for *N. oceanica* IMET1 was from the publication of Li et al. [6] and downloaded as GSE42508 from the Gene Expression Omnibus database. The transcriptome data, which included 9756 genes in FPKM (fragments per kilobase of exon model per million reads mapped) form, was transformed to log2 with the software Perseus [90]. After calculating the respective mean values for three biological replicates, the fold changes between N− and N+ samples were compared (i.e., log2 FPKM (N−/N+)).

### k-shape statistical analysis

A novel algorithm time-series clustering presented by Gravano’s group [41] was used, which relies on a scalable iterative refinement procedure and creates homogeneous and well-separated clusters. The data was normalized both on the cross-correlation and time-series dimensions. To devise a shape-based distance (SBD) measure, a coefficient normalization was used and returned results with values between −1 and 1. The calculation of this distances has been done in in house using the R^®^. For the SBD, we define the highly unsimilar data as its SBD of transcriptome and proteome bigger than upper quartile of all the SBD, which smaller than lower quartile of all the SBD is defined as highly similar.

*Accession Number* The proteome data can be publicly assessed from the PRIDE database via PXD016699.

## Supplementary information

**Additional file 1: Figure** S**1**. PCA analysis of all the samples including three bio-replications.

**Additional file 2: Table S1.** Details of each sub-cluster: K(X)-Function show all the functions of proteins belonging to this sub-cluster and the K(X)-Fold change show all the fold changes of proteins that belong to this sub-cluster.

**Additional file 3: Table** S**2.** LFQ intensities of proteome and FPKM of transcriptome of all identified genes are listed on this table.

**Additional file 4: Table** S**3**. Abbreviation, predicted location and fold changes of key genes on the Calvin cycle, TCA cycle, Kennedy pathway, etc.

**Additional file 5: Figure** S**2**. Storage carbohydrate metabolism proteomics vs. transcriptomics.

**Additional file 6: Figure S3**. *Nannochloropsis oceanica* IMET1 cells after 10 days of N+ and N− treatment, images were taken with Laser confocal fluorescence microscopy. The green color presents the autofluorescence from chloroplast. The red color fluorescence presents the stained liposomes with BODIPY. The orange fluorescence is the overlay from both channels.

**Additional file 7: Figure** S**4**. Growth curve for *N. oceanica* with and without nitrogen starvation.

## Data Availability

All data generated or analyzed during this study are included in its Additional files.

## Abbreviations

BCT: Bicarbonate transporters
CCM: Carbon concentrating mechanisms
CAs: Carbonic anhydrases
PDH: Chloroplast pyruvate dehydrogenase
CS: Citrate synthase
DGAT: Diacylglycerol acyltransferases
EPA: Eicosapentaenoic acid
FDR: False discovery rate
FPKM: Fragments per kilobase of exon model per million reads mapped
GAP: Glyceraldehyde 3-phosphate
GAPDH: Glyceraldehyde 3-phosphate dehydrogenase
GPAT: Glycerol-3-phosphate acyltransferase
KAS: Ketoacyl synthase
LFQ: Label-free relative quantification
LHC: Light-harvesting complex
LDSP: Lipid body surface protein
LC-FACS: Long-chain fatty acyl-CoA synthetase
LPAAT: Lysophosphatidic acid acetyltransferase
MDH: Malate dehydrogenase
ME: NAD(P)-malic enzyme
NL: Neutral lipids
OAA: Oxaloacetate
PEP: Phosphoenolpyruvate
PEPCK: Phosphoenolpyruvate carboxykinase
PEPC: Phosphoenolpyruvate carboxylase
PDAT: Phospholipid diacylglycerol acyltransferase
PL: Polar lipids
PYR: Pyruvate
PDHC: Pyruvate dehydrogenase complex
PK: Pyruvate kinase
PPDK: Pyruvate phosphate dikinase
SBD: Shape-based distance
SDH: Succinate dehydrogenase
TF: Transcription factors
TL: Transketolase
TAG: Triacylglycerides
TPI: Triosephosphate isomerase

## References

[1] Sukenik A, Carmeli Y, Berner T (1989). Regulation of fatty-acid composition by irradiance level in the eustigmatophyte *Nannochloropsis* Sp. J Phycol.

[2] Boussiba S, Vonshak A, Cohen Z, Avissar Y, Richmond A (1987). Lipid and biomass production by the halotolerant microalga *Nannochloropsis salina*. Biomass.

[3] Rodolfi L, Chini Zittelli G, Bassi N, Padovani G, Biondi N, Bonini G, Tredici MR (2009). Microalgae for oil: strain selection, induction of lipid synthesis and outdoor mass cultivation in a low-cost photobioreactor. Biotechnol Bioeng.

[4] Radakovits R, Jinkerson RE, Darzins A, Posewitz MC (2010). Genetic engineering of algae for enhanced biofuel production. Eukaryot Cell.

[5] Wang D, Lu Y, Huang H, Xu J (2012). Establishing oleaginous microalgae research models for consolidated bioprocessing of solar energy. Adv Biochem Eng Biotechnol.

[6] Li J, Han D, Wang D, Ning K, Jia J, Wei L, Jing X, Huang S, Chen J, Li Y (2014). Choreography of transcriptomes and lipidomes of nannochloropsis reveals the mechanisms of oil synthesis in microalgae. Plant Cell.

[7] Chapman KD, Ohlrogge JB (2012). Compartmentation of triacylglycerol accumulation in plants. J Biol Chem.

[8] Chen JE, Smith AG (2012). A look at diacylglycerol acyltransferases (DGATs) in algae. J Biotechnol.

[9] Wang D, Ning K, Li J, Hu J, Han D, Wang H, Zeng X, Jing X, Zhou Q, Su X (2014). Nannochloropsis genomes reveal evolution of microalgal oleaginous traits. PLoS Genet.

[10] Vieler A, Wu G, Tsai CH, Bullard B, Cornish AJ, Harvey C, Reca IB, Thornburg C, Achawanantakun R, Buehl CJ (2012). Genome, functional gene annotation, and nuclear transformation of the heterokont oleaginous alga *Nannochloropsis oceanica* CCMP1779. PLoS Genet.

[11] Radakovits R, Jinkerson RE, Fuerstenberg SI, Tae H, Settlage RE, Boore JL, Posewitz MC (2012). Draft genome sequence and genetic transformation of the oleaginous alga *Nannochloropis gaditana*. Nat Commun.

[12] Corteggiani Carpinelli E, Telatin A, Vitulo N, Forcato C, D’Angelo M, Schiavon R, Vezzi A, Giacometti GM, Morosinotto T, Valle G (2014). Chromosome scale genome assembly and transcriptome profiling of *Nannochloropsis gaditana* in nitrogen depletion. Mol Plant.

[13] Zheng MG, Tian JH, Yang GP, Zheng L, Chen GG, Chen JL, Wang B (2013). Transcriptome sequencing, annotation and expression analysis of *Nannochloropsis* sp. at different growth phases. Gene.

[14] Jia J, Han D, Gerken HG, Li Y, Sommerfeld M, Hu Q, Xu J (2015). Molecular mechanisms for photosynthetic carbon partitioning into storage neutral lipids in *Nannochloropsis oceanica* under nitrogen-depletion conditions. Algal Res.

[15] Hu J, Wang D, Li J, Jing G, Ning K, Xu J (2014). Genome-wide identification of transcription factors and transcription-factor binding sites in oleaginous microalgae Nannochloropsis. Sci Rep.

[16] He Y, Zhang P, Huang S, Wang T, Ji Y, Xu J (2017). Label-free, simultaneous quantification of starch, protein and triacylglycerol in single microalgal cells. Biotechnol Biofuels.

[17] Ma X, Liu J, Liu B, Chen T, Yang B, Chen F (2016). Physiological and biochemical changes reveal stress-associated photosynthetic carbon partitioning into triacylglycerol in the oleaginous marine alga *Nannochloropsis oculata*. Algal Res.

[18] Han D, Jia J, Li J, Sommerfeld M, Xu J, Hu Q (2017). Metabolic remodeling of membrane glycerolipids in the microalga *Nannochloropsis oceanica* under Nitrogen Deprivation. Front Marine Sci.

[19] Tran N-AT, Padula MP, Evenhuis CR, Commault AS, Ralph PJ, Tamburic B (2016). Proteomic and biophysical analyses reveal a metabolic shift in nitrogen deprived *Nannochloropsis oculata*. Algal Res.

[20] Dong HP, Williams E, Wang DZ, Xie ZX, Hsia RC, Jenck A, Halden R, Li J, Chen F, Place AR (2013). Responses of *Nannochloropsis oceanica* IMET1 to long-term nitrogen starvation and recovery. Plant Physiol.

[21] Gargouri M, Park JJ, Holguin FO, Kim MJ, Wang H, Deshpande RR, Shachar-Hill Y, Hicks LM, Gang DR (2015). Identification of regulatory network hubs that control lipid metabolism in Chlamydomonas reinhardtii. J Exp Bot.

[22] Park JJ, Wang H, Gargouri M, Deshpande RR, Skepper JN, Holguin FO, Juergens MT, Shachar-Hill Y, Hicks LM, Gang DR (2015). The response of Chlamydomonas reinhardtii to nitrogen deprivation: a systems biology analysis. Plant J.

[23] Xiao Y, Zhang J, Cui J, Feng Y, Cui Q (2013). Metabolic profiles of *Nannochloropsis oceanica* IMET1 under nitrogen-deficiency stress. Bioresour Technol.

[24] Ji Y, He Y, Cui Y, Wang T, Wang Y, Li Y, Huang WE, Xu J (2014). Raman spectroscopy provides a rapid, non-invasive method for quantitation of starch in live, unicellular microalgae. Biotechnol J.

[25] Wei L, Shen C, El Hajjami M, You W, Wang Q, Zhang P, Ji Y, Hu H, Hu Q, Poetsch A (2019). Knockdown of carbonate anhydrase elevates Nannochloropsis productivity at high CO2 level. Metab Eng.

[26] Wang Q, Lu Y, Xin Y, Wei L, Huang S, Xu J (2016). Genome editing of model oleaginous microalgae Nannochloropsis spp by CRISPR/Cas9. Plant J.

[27] Wei L, Xin Y, Wang Q, Yang J, Hu H, Xu J (2016). RNAi-based targeted gene-knockdown in the model oleaginous microalgae *Nannochloropsis oceanica*. Plant J.

[28] Wang T, Ji Y, Wang Y, Jia J, Li J, Huang S, Han D, Hu Q, Huang WE, Xu J (2014). Quantitative dynamics of triacylglycerol accumulation in microalgae populations at single-cell resolution revealed by Raman microspectroscopy. Biotechnol Biofuels.

[29] Wijffels RH, Barbosa MJ (2010). An outlook on microalgal biofuels. Science.

[30] Lee SY, Cho JM, Chang YK, Oh YK (2017). Cell disruption and lipid extraction for microalgal biorefineries: a review. Bioresour Technol.

[31] Wei L, El Hajjami M, Shen C, You W, Lu Y, Li J, Jing X, Hu Q, Zhou W, Poetsch A (2019). Transcriptomic and proteomic responses to very low CO2 suggest multiple carbon concentrating mechanisms in *Nannochloropsis oceanica*. Biotechnol Biofuels.

[32] Wei L, Wang Q, Xin Y, Lu Y, Xu J (2017). Enhancing photosynthetic biomass productivity of industrial oleaginous microalgae by overexpression of RuBisCO activase. Algal Res.

[33] Xin Y, Shen C, She Y, Chen H, Wang C, Wei L, Yoon K, Han D, Hu Q, Xu J (2019). Biosynthesis of triacylglycerol molecules with a tailored PUFA profile in industrial microalgae. Molecular plant.

[34] Xin Y, Lu Y, Lee YY, Wei L, Jia J, Wang Q, Wang D, Bai F, Hu H, Hu Q (2017). Producing designer oils in industrial microalgae by rational modulation of co-evolving type-2 diacylglycerol acyltransferases. Mol Plant.

[35] Poliner E, Farre EM, Benning C (2018). Advanced genetic tools enable synthetic biology in the oleaginous microalgae *Nannochloropsis* sp. Plant Cell Rep.

[36] Reinfelder JR (2011). Carbon concentrating mechanisms in eukaryotic marine phytoplankton. Ann Rev Mar Sci.

[37] Tyanova S, Temu T, Cox J (2016). The MaxQuant computational platform for mass spectrometry-based shotgun proteomics. Nat Protoc.

[38] Pacholewska A (2017). Loget”-a Uniform Differential Expression Unit to Replace” logFC” and” log2FC. Matters.

[39] States DJ, Gish W (1994). Combined use of sequence similarity and codon bias for coding region identification. J Comput Biol.

[40] Abraham PE, Yin H, Borland AM, Weighill D, Lim SD, De Paoli HC, Engle N, Jones PC, Agh R, Weston DJ (2016). Transcript, protein and metabolite temporal dynamics in the CAM plant Agave. Nat Plants.

[41] Paparrizos J, Gravano L: k-Shape. In: *Proceedings of the 2015 ACM SIGMOD International Conference on Management of Data* - *SIGMOD ‘15*. 2015. p 1855–1870.

[42] Wang H, Gau B, Slade WO, Juergens M, Li P, Hicks LM (2014). The global phosphoproteome of Chlamydomonas reinhardtii reveals complex organellar phosphorylation in the flagella and thylakoid membrane. Mol Cell Proteom.

[43] Emanuelsson O, Nielsen H, Von Heijne G (1999). ChloroP, a neural network-based method for predicting chloroplast transit peptides and their cleavage sites. Protein Sci.

[44] Gee CW, Niyogi KK (2017). The carbonic anhydrase CAH1 is an essential component of the carbon-concentrating mechanism in *Nannochloropsis oceanica*. Proc Natl Acad Sci USA.

[45] Almagro Armenteros JJ, Tsirigos KD, Sonderby CK, Petersen TN, Winther O, Brunak S, von Heijne G, Nielsen H (2019). SignalP 5.0 improves signal peptide predictions using deep neural networks. Nat Biotechnol.

[46] Almagro Armenteros JJ, Salvatore M, Emanuelsson O, Winther O, von Heijne G, Elofsson A, Nielsen H (2019). Detecting sequence signals in targeting peptides using deep learning. Life Sci Alliance.

[47] Kang F, Rawsthorne S (1996). Metabolism of glucose-6-phosphate and utilization of multiple metabolites for fatty acid synthesis by plastids from developing oilseed rape embryos. Planta.

[48] Alonso AP, Goffman FD, Ohlrogge JB, Shachar-Hill Y (2007). Carbon conversion efficiency and central metabolic fluxes in developing sunflower (*Helianthus annuus* L.) embryos. Plant J.

[49] Reverdatto S, Beilinson V, Nielsen NC (1999). A multisubunit acetyl coenzyme A carboxylase from soybean. Plant Physiol.

[50] Li D-W, Balamurugan S, Yang Y-F, Zheng J-W, Huang D, Zou L-G, Yang W-D, Liu J-S, Guan Y, Li H-Y (2019). Transcriptional regulation of microalgae for concurrent lipid overproduction and secretion. Sci Adv.

[51] Gargiulo CE, Stuhlsatz-Krouper SM, Schaffer JE (1999). Localization of adipocyte long-chain fatty acyl-CoA synthetase at the plasma membrane. J Lipid Res.

[52] Suen Y, Hubbard JS, Holzer G, Tornabene TG (1987). Total Lipid Production of the Green-Alga *Nannochloropsis* sp. Qii under Different Nitrogen Regimes. J Phycol.

[53] Zhang Y, Wu H, Sun M, Peng Q, Li A (2018). Photosynthetic physiological performance and proteomic profiling of the oleaginous algae *Scenedesmus acuminatus* reveal the mechanism of lipid accumulation under low and high nitrogen supplies. Photosynth Res.

[54] Janssen JH, Kastenhofer J, de Hoop JA, Lamers PP, Wijffels RH, Barbosa MJ (2018). Effect of nitrogen addition on lipid productivity of nitrogen starved *Nannochloropsis gaditana*. Algal Res.

[55] Ma X, Yao L, Yang B, Lee YK, Chen F, Liu J (2017). RNAi-mediated silencing of a pyruvate dehydrogenase kinase enhances triacylglycerol biosynthesis in the oleaginous marine alga *Nannochloropsis salina*. Sci Rep.

[56] Hu Q, Sommerfeld M, Jarvis E, Ghirardi M, Posewitz M, Seibert M, Darzins A (2008). Microalgal triacylglycerols as feedstocks for biofuel production: perspectives and advances. Plant J.

[57] Simionato D, Block MA, La Rocca N, Jouhet J, Marechal E, Finazzi G, Morosinotto T (2013). The response of *Nannochloropsis gaditana* to nitrogen starvation includes de novo biosynthesis of triacylglycerols, a decrease of chloroplast galactolipids, and reorganization of the photosynthetic apparatus. Eukaryot Cell.

[58] Park S, Steen CJ, Lyska D, Fischer AL, Endelman B, Iwai M, Niyogi KK, Fleming GR (2019). Chlorophyll-carotenoid excitation energy transfer and charge transfer in *Nannochloropsis oceanica* for the regulation of photosynthesis. Proc Natl Acad Sci USA.

[59] Vello V, Chu W-L, Lim P-E, Majid NA, Phang S-M (2018). Metabolomic profiles of tropical Chlorella species in response to physiological changes during nitrogen deprivation. J Appl Phycol.

[60] Kotzsch A, Pawolski D, Milentyev A, Shevchenko A, Scheffel A, Poulsen N, Shevchenko A, Kroger N (2016). Biochemical composition and assembly of biosilica-associated insoluble organic matrices from the diatom *Thalassiosira pseudonana*. J Biol Chem.

[61] Basso S, Simionato D, Gerotto C, Segalla A, Giacometti GM, Morosinotto T (2014). Characterization of the photosynthetic apparatus of the Eustigmatophycean *Nannochloropsis gaditana*: evidence of convergent evolution in the supramolecular organization of photosystem I. Biochem Biophys Acta.

[62] Kim JH, Nemson JA, Melis A (1993). Photosystem II reaction center damage and repair in *Dunaliella salina* (Green Alga) (analysis under physiological and irradiance-stress conditions). Plant Physiol.

[63] Mackinder LCM, Chen C, Leib RD, Patena W, Blum SR, Rodman M, Ramundo S, Adams CM, Jonikas MC (2017). A spatial interactome reveals the protein organization of the algal CO_2_-concentrating mechanism. Cell.

[64] Sedelnikova OV, Hughes TE, Langdale JA (2018). Understanding the genetic basis of C4 Kranz anatomy with a view to engineering C3 crops. Annu Rev Genet.

[65] Li-Beisson Y, Thelen JJ, Fedosejevs E, Harwood JL (2019). The lipid biochemistry of eukaryotic algae. Prog Lipid Res.

[66] Rai V, Muthuraj M, Gandhi MN, Das D, Srivastava S (2017). Real-time iTRAQ-based proteome profiling revealed the central metabolism involved in nitrogen starvation induced lipid accumulation in microalgae. Sci Rep.

[67] Dall’Osto L, Cazzaniga S, Guardini Z, Barera S, Benedetti M, Mannino G, Maffei ME, Bassi R (2019). Combined resistance to oxidative stress and reduced antenna size enhance light-to-biomass conversion efficiency in Chlorella vulgaris cultures. Biotechnol Biofuels.

[68] Yoshikawa K, Toya Y, Shimizu H (2017). Metabolic engineering of *Synechocystis* sp PCC 6803 for enhanced ethanol production based on flux balance analysis. Bioprocess Biosyst Eng.

[69] Chen JW, Liu WJ, Hu DX, Wang X, Balamurugan S, Alimujiang A, Yang WD, Liu JS, Li HY (2017). Identification of a malonyl CoA-acyl carrier protein transacylase and its regulatory role in fatty acid biosynthesis in oleaginous microalga *Nannochloropsis oceanica*. Biotechnol Appl Biochem.

[70] Li Z, Meng T, Ling X, Li J, Zheng C, Shi Y, Chen Z, Li Z, Li Q, Lu Y (2018). Overexpression of Malonyl-CoA: ACP Transacylase in *Schizochytrium* sp. to Improve polyunsaturated fatty acid production. J Agric Food Chem.

[71] Ohlrogge J, Browse J (1995). Lipid biosynthesis. Plant Cell.

[72] Fan J, Yan C, Andre C, Shanklin J, Schwender J, Xu C (2012). Oil accumulation is controlled by carbon precursor supply for fatty acid synthesis in *Chlamydomonas reinhardtii*. Plant Cell Physiol.

[73] Schwender J, Shachar-Hill Y, Ohlrogge JB (2006). Mitochondrial metabolism in developing embryos of Brassica napus. J Biol Chem.

[74] Matos AR, d’Arcy-Lameta A, Franca M, Petres S, Edelman L, Kader J, Zuily-Fodil Y, Pham-Thi AT (2001). A novel patatin-like gene stimulated by drought stress encodes a galactolipid acyl hydrolase. FEBS Lett.

[75] Yang WY, Devaiah SP, Pan XQ, Isaac G, Welti R, Wang XM (2007). AtPLAI is an acyl hydrolase involved in basal jasmonic acid production and Arabidopsis resistance to Botrytis cinerea. J Biol Chem.

[76] Miller R, Wu G, Deshpande RR, Vieler A, Gartner K, Li X, Moellering ER, Zauner S, Cornish AJ, Liu B (2010). Changes in transcript abundance in *Chlamydomonas reinhardtii* following nitrogen deprivation predict diversion of metabolism. Plant Physiol.

[77] Varshavsky A (2019). N-degron and C-degron pathways of protein degradation. Proc Natl Acad Sci USA.

[78] Ratledge C (2004). Fatty acid biosynthesis in microorganisms being used for Single Cell Oil production. Biochimie.

[79] Janssen JH, Lamers PP, de Vos RCH, Wijffels RH, Barbosa MJ (2019). Translocation and de novo synthesis of eicosapentaenoic acid (EPA) during nitrogen starvation in *Nannochloropsis gaditana*. Algal Res.

[80] Janssen JH, Wijffels RH, Barbosa MJ (2019). Lipid Production in *Nannochloropsis gaditana* during Nitrogen Starvation. Biology (Basel).

[81] Wan C, Bai F-W, Zhao X-Q (2013). Effects of nitrogen concentration and media replacement on cell growth and lipid production of oleaginous marine microalga *Nannochloropsis oceanica* DUT01. Biochem Eng J.

[82] Jeong SW, Nam SW, HwangBo K, Jeong WJ, Jeong BR, Chang YK, Park YI (2017). Transcriptional regulation of cellulose biosynthesis during the early phase of nitrogen deprivation in *Nannochloropsis salina*. Sci Rep.

[83] Li Y, Han D, Hu G, Dauvillee D, Sommerfeld M, Ball S, Hu Q (2010). Chlamydomonas starchless mutant defective in ADP-glucose pyrophosphorylase hyper-accumulates triacylglycerol. Metab Eng.

[84] Huang W, Haferkamp I, Lepetit B, Molchanova M, Hou S, Jeblick W, Rio Bartulos C, Kroth PG (2018). Reduced vacuolar beta-1,3-glucan synthesis affects carbohydrate metabolism as well as plastid homeostasis and structure in Phaeodactylum tricornutum. Proc Natl Acad Sci USA.

[85] Aucoin HR, Gardner J, Boyle NR (2016). Omics in chlamydomonas for biofuel production. Sub-Cell Biochem.

[86] Su J, Yang X, Zheng T, Hong H (2007). An efficient method to obtain axenic cultures of *Alexandrium tamarense*—a PSP-producing dinoflagellate. J Microbiol Methods.

[87] Abida H, Dolch LJ, Mei C, Villanova V, Conte M, Block MA, Finazzi G, Bastien O, Tirichine L, Bowler C (2015). Membrane glycerolipid remodeling triggered by nitrogen and phosphorus starvation in *Phaeodactylum tricornutum*. Plant Physiol.

[88] Cox J, Neuhauser N, Michalski A, Scheltema RA, Olsen JV, Mann M (2011). Andromeda: a peptide search engine integrated into the MaxQuant environment. J Proteome Res.

[89] Cox J, Mann M (2008). MaxQuant enables high peptide identification rates, individualized p.p.b.-range mass accuracies and proteome-wide protein quantification. Nat Biotechnol.

[90] Tyanova S, Temu T, Sinitcyn P, Carlson A, Hein MY, Geiger T, Mann M, Cox J (2016). The Perseus computational platform for comprehensive analysis of (prote)omics data. Nat Methods.

[91] Petersen TN, Brunak S, von Heijne G, Nielsen H (2011). SignalP 40: discriminating signal peptides from transmembrane regions. Nat Methods.

