# Supplementary figures and images for: Integration of proteome and transcriptome refines key molecular processes underlying oil production in Nannochloropsis oceanica
Source: Biotechnol Biofuels. 2020 Jun 18;13:109. doi: 10.1186/s13068-020-01748-2 (PMC7302151; doi:10.1186/s13068-020-01748-2)

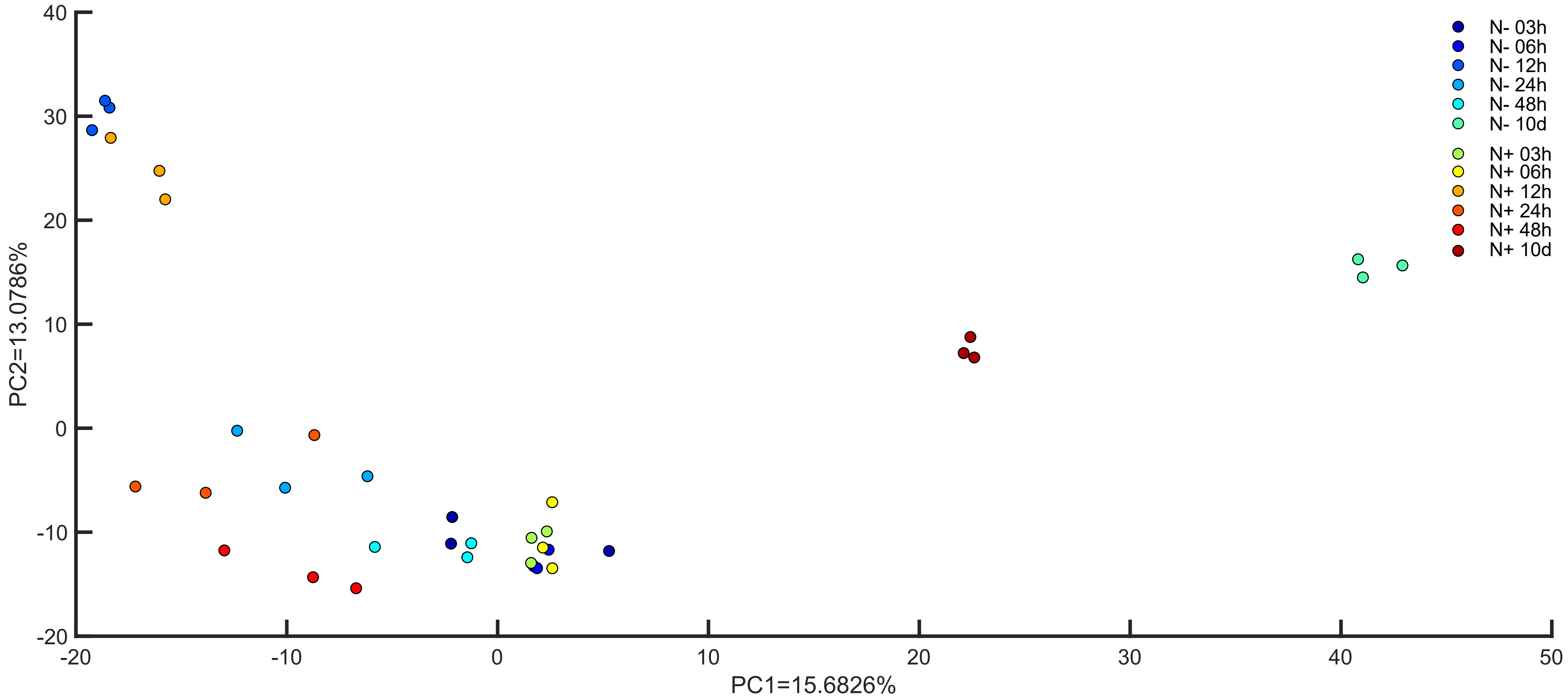

Supplement: Supplementary file 1 — Additional file 1: Figure S1. PCA analysis of all the samples including three bio-replications. [file 13068_2020_1748_MOESM1_ESM.pdf]

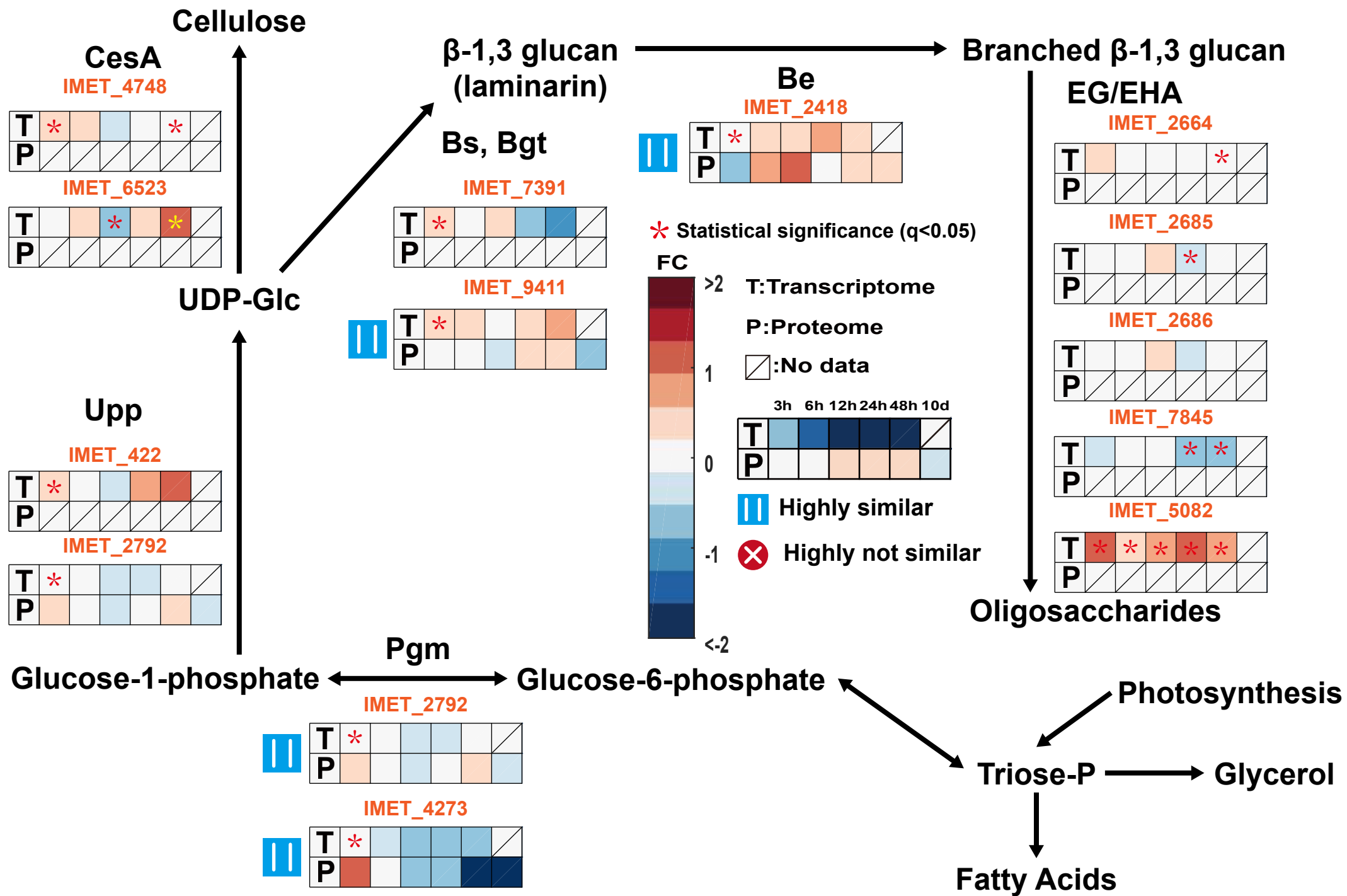

Supplement: Supplementary file 5 — Additional file 5: Figure S2. Storage carbohydrate metabolism proteomics vs. transcriptomics. [file 13068_2020_1748_MOESM5_ESM.pdf]

**N+**

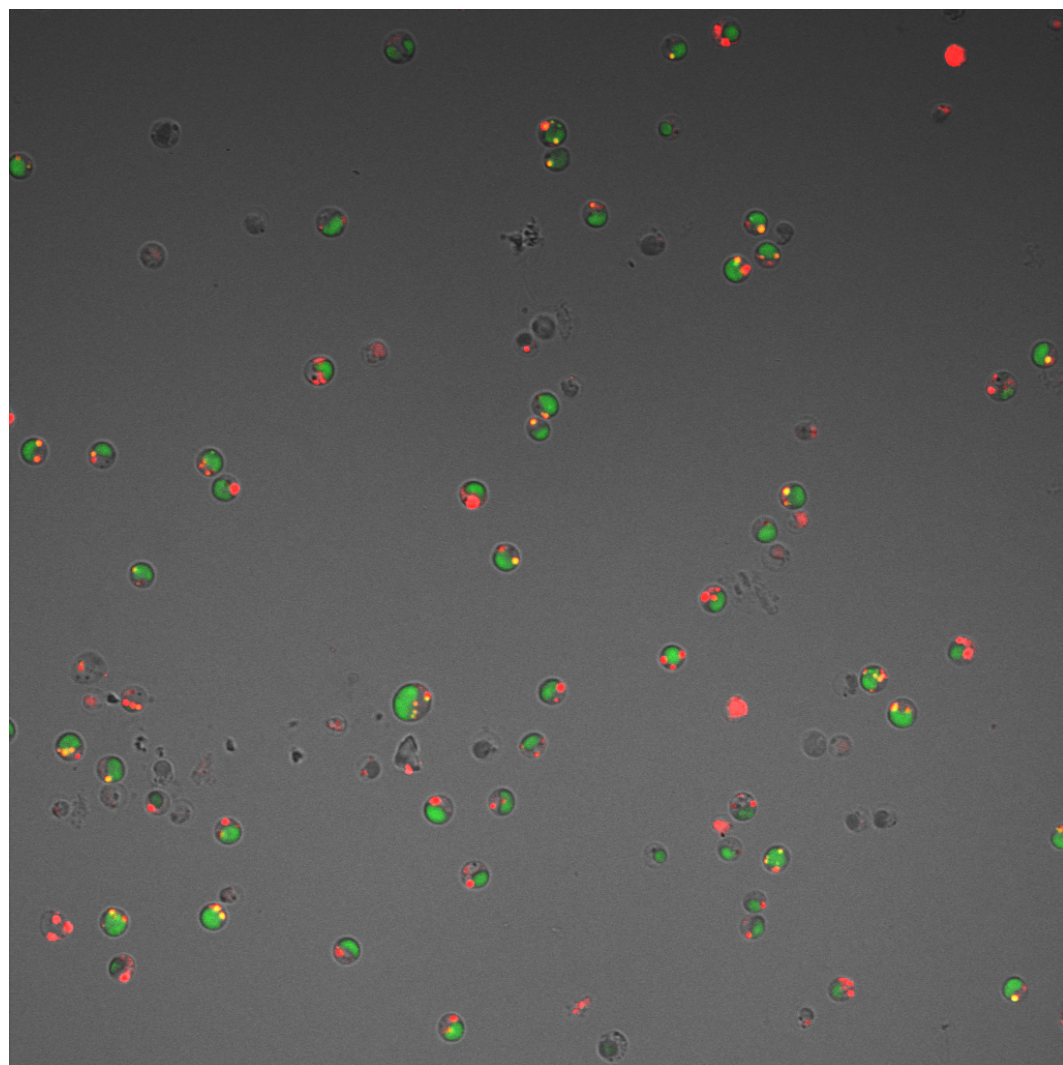

**N-**

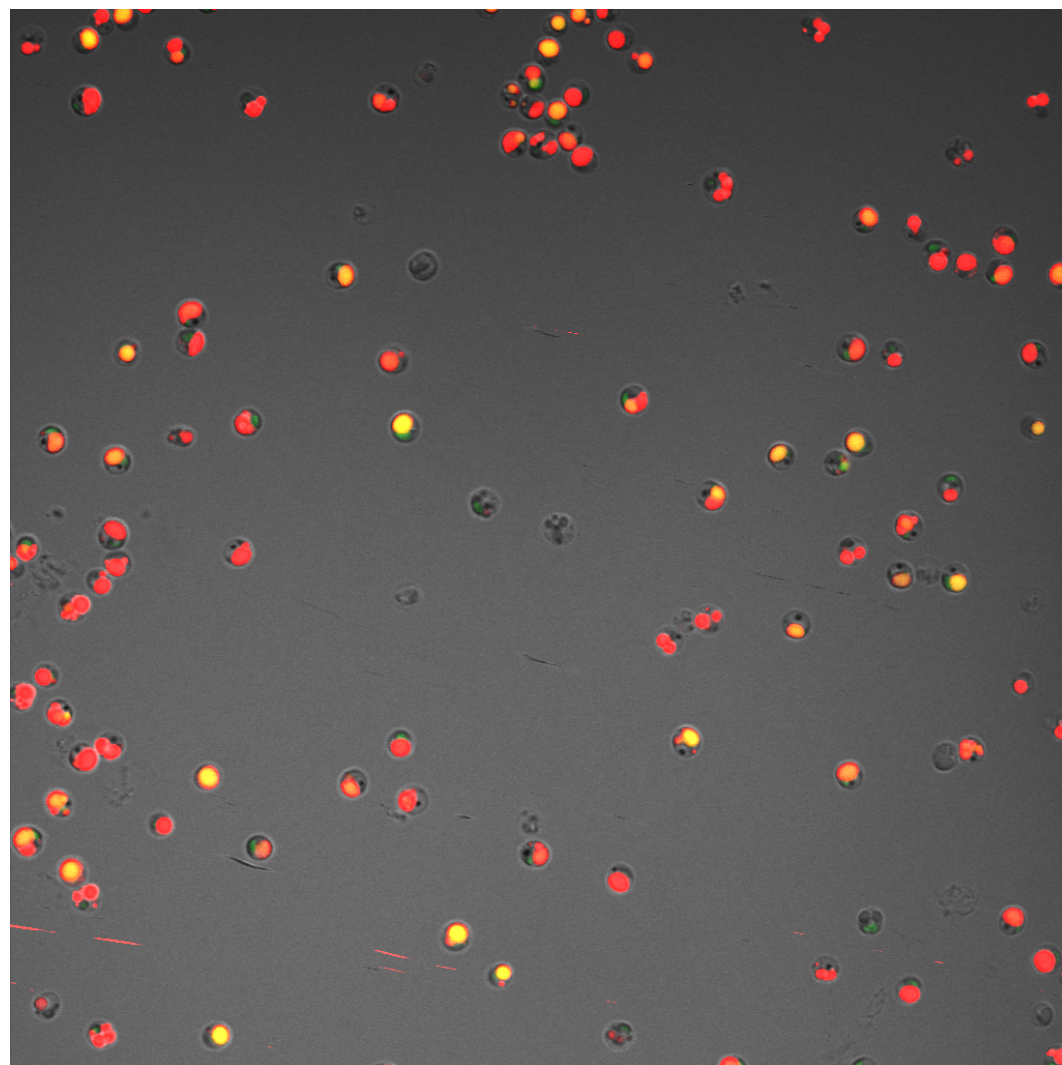

Supplement: Supplementary file 6 — Additional file 6: Figure S3. Nannochloropsis oceanica IMET1 cells after 10 days of N+ and N− treatment, images were taken with Laser confocal fluorescence microscopy. The green color presents the autofluorescence from chloroplast. The red color fluorescence presents the stained liposomes with BODIPY. The orange fluorescence is the overlay from both channels. [file 13068_2020_1748_MOESM6_ESM.pdf]

# Growth Curve

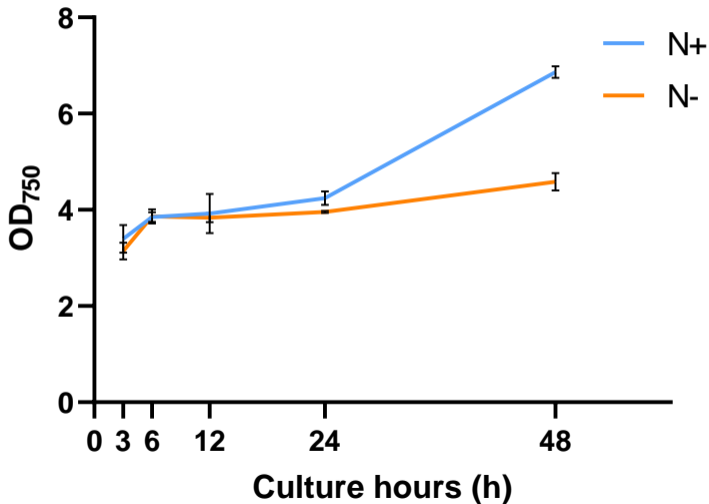

Supplement: Supplementary file 7 — Additional file 7: Figure S4. Growth curve for N. oceanica with and without nitrogen starvation. [file 13068_2020_1748_MOESM7_ESM.pdf]
